# Electrochemical oscillation triggered by the doped Mn ions in spinel Li_4_Ti_5_O_12_

**DOI:** 10.1039/d6sc05042e

**Published:** 2026-07-21

**Authors:** Wenting Ji, Jie Bai, Yuqin Huang, De Li, Yong Chen, Haoshen Zhou

**Affiliations:** a State Key Lab of Tropic Ocean Engineering Materials and Materials Evaluation, School of Materials Science and Engineering, Hainan Provincial Key Laboratory of Research on Utilization of Si–Zr–Ti Resource, Hainan University Haikou 570228 China lidehainu@hainanu.edu.cn; b School of Materials and Energy, Foshan University Foshan 528000 China ychen2002@163.com; c Department of Applied Chemistry, School of Chemistry and Chemical Engineering, Chongqing University Chongqing 401331 China; d Center of Energy Storage Materials & Technology, College of Engineering and Applied Sciences, Jiangsu Key Laboratory of Artificial Functional Materials, National Laboratory of Solid-State Microstructures and Collaborative Innovation Center of Advanced Microstructures, Nanjing University Nanjing 210093 China

## Abstract

Phase-transition kinetics is an important concern in the wide application of Li-ion batteries, while its in-depth understanding is still limited by the complexity of real electrode systems. As a derivative phenomenon, electrochemical oscillation is closely coupled with the underlying phase-transition kinetics, providing a direct and sensitive probe to track kinetic variations. Herein, a valence-engineering strategy is presented to modulate the phase-transition kinetics of Mn-doped Li_4_Ti_5_O_12_ (LMTO), which can be directly manifested through the characteristic evolution of electrochemical oscillation behaviour. Actually, LMTO-Ar (sintering under Ar) exhibits enhanced oscillatory amplitude during de-lithiation, whereas LMTO-O_2_ (sintering under O_2_) triggers oscillation at the onset of lithiation, which can be interpreted using the split overpotentials of nucleation and phase transition, owing to the high overpotential of the nucleation step and the hysteresis of the phase-transition step in LMTO. Additionally, the electrochemical oscillation is quantitatively reproduced with a million-particle electrode through tailoring the nucleation and phase-transition kinetics. Therefore, this investigation provides profound insights into the origin of electrochemical oscillation and establishes a generalizable kinetics–oscillation correlation framework for advancing the rational design of phase-transition electrode materials.

## Abstract 2

Mn dopants regulate nucleation and phase-transition kinetics to control electrochemical oscillations in electrodes.

## Graphical abstract

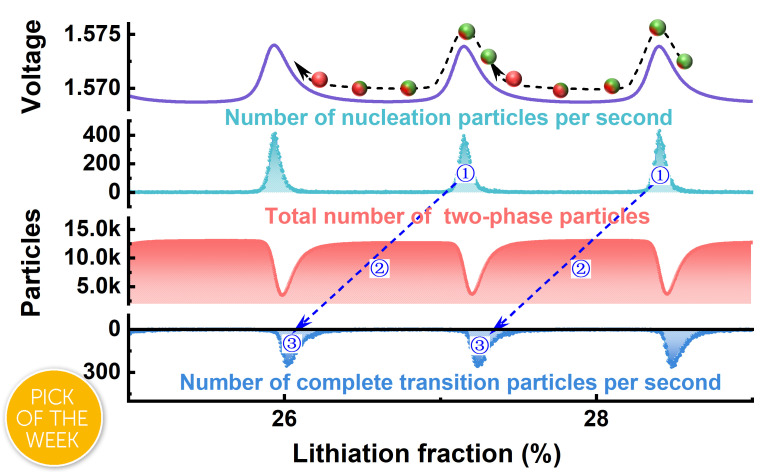

## Introduction

Nucleation and phase transitions underpin the functionality of a broad range of advanced materials, from perovskites,^1^ microelectronics,^2^ and energy catalysts^3^ to battery electrodes.^4–8^ Rechargeable Li-ion batteries (LIBs) have been widely applied in electric vehicles, mobile electronics, smart grids, *etc.*, owing to their long cycle life, excellent rate capability and inherent safety.^9–12^ Limited by the kinetics of nucleation, phase transitions, *etc.*, LIBs inherently operate under non-equilibrium conditions during charge–discharge cycling yielding intriguing physical and chemical properties, such as the memory effect, voltage hysteresis, and electrochemical oscillation. Thus, the underlying kinetics is significant for battery applications and fundamental studies but is rarely elucidated experimentally.

The random reactions of multi-particle electrodes can result in a smooth voltage plateau without information on single-particle reactions,^13,14^ leading to a long-standing challenge to decipher the behaviour of practical particles in complex electrode systems. In some modified Li_4_Ti_5_O_12_ (LTO) anodes, the multi-particle electrode would undergo a discrete reaction and induce an electrochemical oscillation, retaining the single-particle information. There are some studies about electrochemical oscillation in several two-phase materials. Zhou's group reported for the first time periodic voltage and current fluctuation phenomena (electrochemical oscillation) for surface-modified LTO anodes.^15^ A similar electrochemical oscillation was also observed in another two-phase material of spinel LiCrTiO_4_, which was synthesized by using spray-drying-sintering methods.^16^ Huang *et al.* introduced Al–ZnO surface coatings on LTO thin-film anodes to manipulate the solid-electrolyte interphase, resulting in the suppression of voltage oscillation.^17^ And Lim *et al.* reported that atmosphere-treated LTO with surface oxygen vacancies exhibits both memory effects and electrochemical oscillation.^18^ Besides, Que *et al.* recently observed analogous oscillation behaviour in low-temperature Na-ion batteries (SIBs) by tuning the solvation energy barrier of Na-ions.^19^ As-reported electrochemical oscillations have been predominantly observed in two-phase materials, which involve phase transitions at the micro-nanoscale, so it is still poorly understood how the multi-particle electrode induces macroscopic voltage oscillation.

Besides surface modification, ionic doping is an effective strategy to regulate the electrochemical properties of electrode materials in LIBs. Zhou *et al.* employed a heteroatom doping strategy with boron for silicon and sulfur for phosphorus to reduce activation energies and accelerate redox kinetics during lithiation/de-lithiation processes.^20^ The Huang group reported that introducing elements with low-energy 3d orbitals could expand the energy gap between the e_g_ orbitals and Fermi energy of Na toward the improved intrinsic electronic conductivity of Na_2_Fe_0.45_Mn_0.55_PO_4_F.^21^ And Li *et al.* designed a reduced atmosphere during the annealing process to facilitate low-valence and high-valence Mn-based materials.^22^ Inspired by these studies, we present a valence-engineering strategy to modulate the Mn-doped LTO (LMTO) and found that LMTO-Ar (sintering under Ar) with a low valence and LMTO-O_2_ (sintering under O_2_) with a high valence of Mn exhibited exclusively charge oscillation or discharge oscillation, respectively. Combining with the numerical simulation results, we proposed that the electrochemical oscillation can be attributed to the high overpotential of the nucleation step and the hysteresis of the phase-transition step in Mn-doped LTO, which can be regulated by the valence states and lattice sites of Mn ions. Thereby, this study not only offers a viable strategy for regulating electrochemical oscillation through ion doping, but also provides an innovative perspective on understanding the electrode kinetics within two-phase reactions.

## Results and discussion

### Triggering mechanism of electrochemical oscillation

**Fig. 1:**
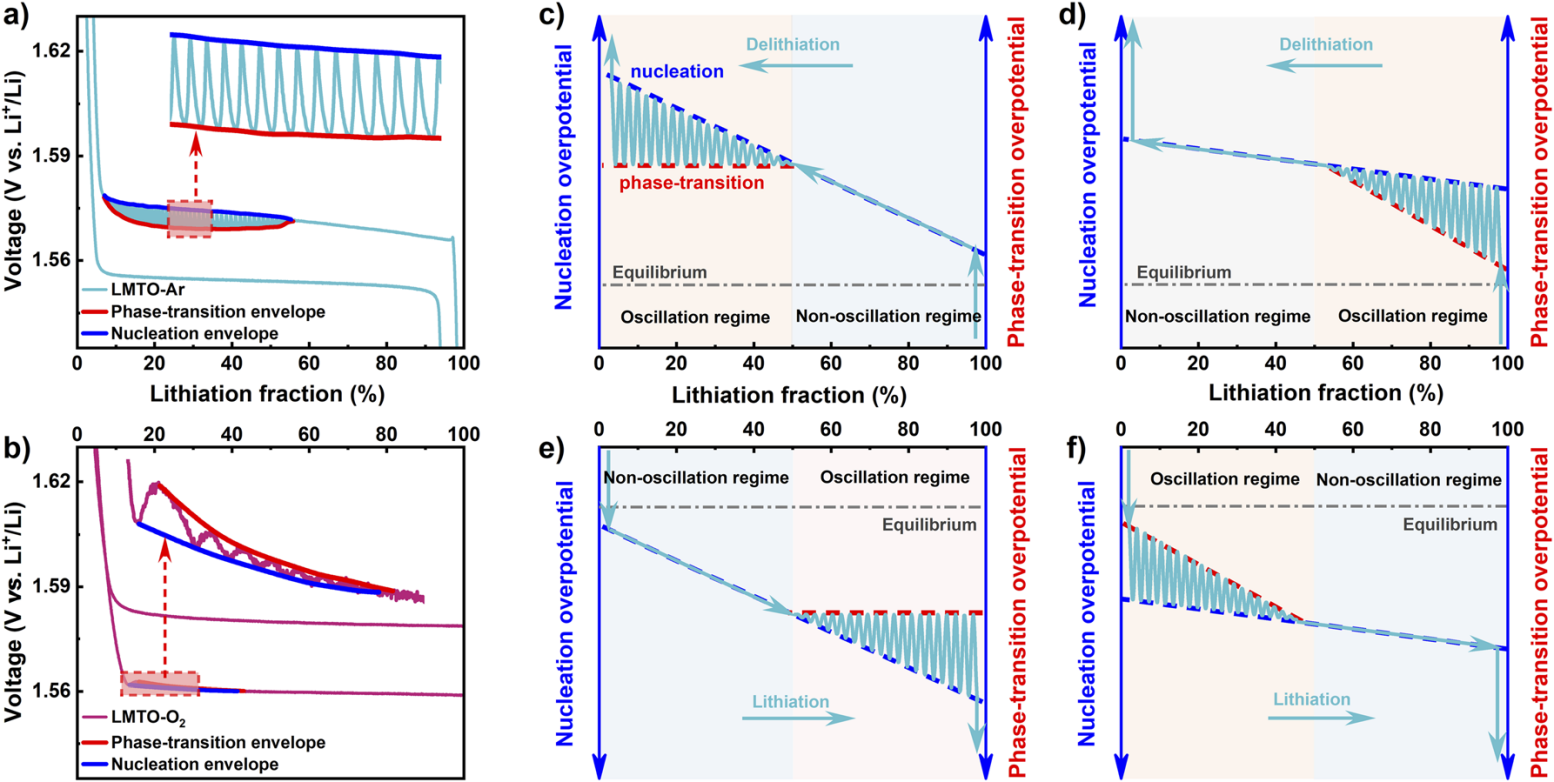
Galvanostatic charge/discharge curves at 0.1C of (a) LMTO-Ar and (b) LMTO-O_2_, in which the peak and valley of the oscillatory regime were delineated by blue and red lines. (c–f) Schematic mechanism for triggering electrochemical oscillation behaviour by alternatively switching between nucleation and phase transition. Oscillation behaviour occurs at the back (c) and front (d) ends of the de-lithiation voltage plateau and the back (e) and front (f) ends of the lithiation voltage plateau.

Utilizing Ar and O_2_ atmospheres during sintering processes, we synthesized different Mn-doped LTO samples, denoted as LMTO-Ar and LMTO-O_2_. As shown in Fig. 1a, LMTO-Ar shows lots of “periodicity waves” in the de-lithiation voltage plateau, *i.e.*, the electrochemical oscillation behaviour, while in the lithiation plateau, the phenomenon is not observed. In contrast, for the LMTO-O_2_ electrode, the electrochemical oscillation phenomenon emerges in the onset region of the lithiation voltage platform, instead of the de-lithiation voltage plateau, as shown in Fig. 1b. To analyse the periodic oscillatory behaviour, the high and low overpotentials within each period are defined as peak and valley, respectively, and we delineated the peak (upper envelope) and valley (lower envelope) of all periods within the oscillatory region, respectively. Previous studies have established that two-phase electrode materials (Li_4_Ti_5_O_12_,^23^ LiFePO_4_,^24^ LiCrTiO_4_, *etc.*) accommodate and release Li ions through nucleation and phase-transition processes. Active particles first form supercritical embryos of the new phase through a nucleation process, which requires overcoming a nucleation barrier with a relatively high overpotential; therefore this step corresponds to the upper envelope. After nucleation, the system enters a two-phase coexistence regime, where the phase separation proceeds with a reduced overpotential, corresponding to the lower envelope. Accordingly, we proposed a schematic scenario to interpret the electrochemical oscillation, as shown in Fig. 1c–f. For the oscillation at the back of the de-lithiation voltage plateau, the overpotential gradually increases in the non-oscillation regime and the overpotentials of nucleation and phase transition split in the oscillation regime, in which the overpotential of phase transition seems to reach a threshold, as shown in Fig. 1c. For the oscillation at the front of the de-lithiation voltage plateau, the overpotential of phase transition is lower than that of nucleation in the oscillation regime, which merge and further increase in the non-oscillation regime, as shown in Fig. 1d. Similar overpotential evolutions are plotted for the oscillation at the back and front of the lithiation voltage plateau in Fig. 1e and f, respectively. Here, the charge oscillation of LMTO-Ar corresponds to the case in Fig. 1c and the discharge oscillation of LMTO-O_2_ corresponds to the case in Fig. 1f. Therefore, the electrochemical oscillation can be interpreted using the split overpotentials of nucleation and phase-transition steps, owing to the high overpotential of the nucleation step and the hysteresis of the phase-transition step in Mn-doped LTO.

### Structural analysis

**Fig. 2:**
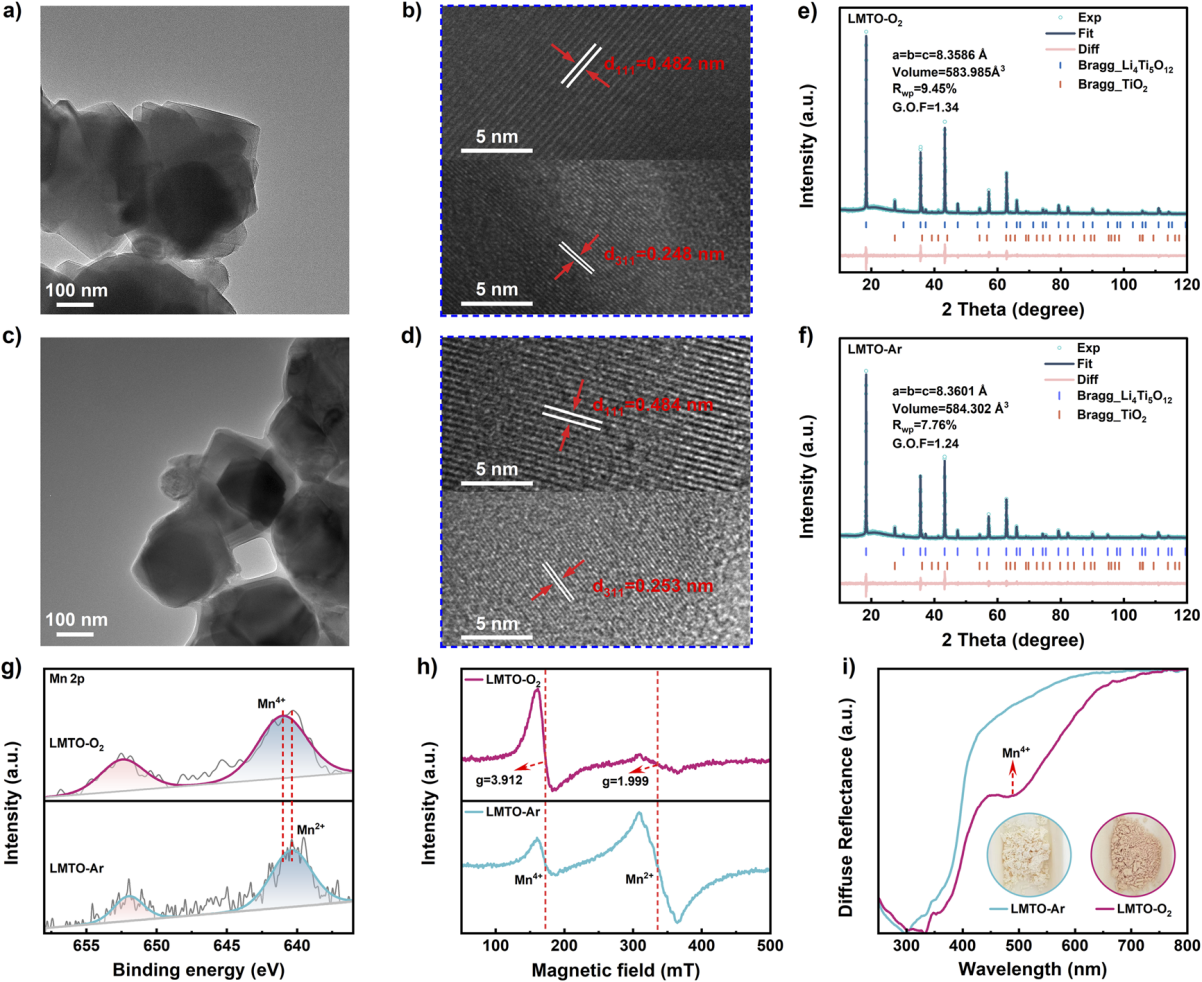
Structure analysis. (a and c) TEM images (b and d) and HRTEM images of LMTO-O_2_ and LMTO-Ar particles. (e and f) Refined XRD patterns of LMTO-O_2_ and LMTO-Ar, weighted profile R-factor agreement factors (*R*_wp_) and goodness of fit (G. O. F.) and lattice parameters are inserted. (g) Mn 2p XPS spectra of LMTO-O_2_ and LMTO-Ar. (h) EPR spectra of LMTO-O_2_ and LMTO-Ar measured at 77 K. (i) UV-vis DRS of LMTO-O_2_ and LMTO-Ar; the insets show the digital photographs of sample powders.

The transmission electron microscopy (TEM) images and scanning electron microscope (SEM) images of as-prepared samples are presented in Fig. 2a, c and S1 (SI). The LTO-O_2_, LMTO-O_2_, LTO-Ar and LMTO-Ar samples have similar morphologies, composed of irregular polyhedra with a sharp surface (no Mn-rich clusters or segregations are observed on the particle surface), and the particle sizes are distributed within a level of a few hundred nanometres. The high-resolution TEM (HRTEM) images reveal uniform lattice fringes of LMTO-O_2_ and LMTO-Ar, with interplanar spacings of 0.248 and 0.482 nm for LMTO-O_2_ (Fig. 2b) and 0.253 and 0.484 nm for LMTO-Ar (Fig. 2d), corresponding to the (311) and (111) planes of spinel LTO, respectively.^25^ All elements are uniformly distributed within the particles of LMTO-O_2_ and LMTO-Ar, as revealed by TEM-EDX element mapping analysis (Fig. S2 and S3).

Fig. S4a displays the XRD patterns of all samples, which are well-indexed as the spinel Li_4_Ti_5_O_12_ structure with a space group of *Fd*3̄*m* (JCPDS No. 49-0207).^26,27^ TiO_2_ peaks are observed in prepared samples due to the volatilization of the Li element during high-temperature sintering, and the graphite powder is mixed into the samples for calibration. The Li_4_Ti_5_O_12_ phase and the TiO_2_ impurity are also verified using Raman spectra that show identical vibration modes for LTO-O_2_, LMTO-O_2_, LTO-Ar and LMTO-Ar (Fig. S5).^28,29^ Compared with the undoped LTO samples, the (400) peak shifts to lower and higher angles for LMTO-Ar (lattice expansion) and LMTO-O_2_ (lattice contraction), respectively (Fig. S4b). Through Rietveld refinements using GSAS-II software, the lattice parameters and other relevant data are detailed in Tables S1–S4 and the refined XRD patterns are illustrated in Fig. 2e and f. The cell volume (584.302 Å^3^) of LMTO-Ar is slightly larger than that of LMTO-O_2_ (583.985 Å^3^), indicating that the doped Mn ions are successfully incorporated into the Li_4_Ti_5_O_12_ crystal lattice and exhibit different states for the two samples.

The valence information of LMTO-O_2_ and LMTO-Ar samples is further revealed by X-ray photoelectron spectra (XPS) and electron paramagnetic resonance spectroscopy (EPR at 77 K). Fig. S6 and 2 g display the localized XPS spectra of Li, O, Ti and Mn for the LMTO-O_2_ and LMTO-Ar samples. The Mn 2p XPS spectrum of LMTO-O_2_ is located at higher binding energy than that of LMTO-Ar, reflecting the higher Mn valence state obtained by sintering under an oxidative atmosphere.^30^ As shown in Fig. 2h, both LMTO-O_2_ and LMTO-Ar show detectable EPR signals at *g* values of ∼3.912 and ∼1.999, corresponding to Mn^4+^ and Mn^2+^ species, respectively,^31–33^ so the LMTO-O_2_ and LMTO-Ar samples feature the predominant valence states of Mn^4+^ and Mn^2+^, respectively. The valence states are further verified by the ultraviolet-visible diffuse reflectance spectra (UV-vis DRS) as illustrated in Fig. 2i. A characteristic absorption peak at ∼550 nm for LMTO-O_2_ originated from the spin-allowed ^4^A_2g_ → ^4^T_2g_ transition of Mn^4+^, instead of Mn^2+^,^34,35^ and the absorption edge position of LMTO-O_2_ is red-shifted compared with LMTO-Ar, implying superior electrical conductivity, consistent with their distinct powder colors namely bright pink for LMTO-O_2_ and blush white for LMTO-Ar (Fig. 2i inset). Combining with the XRD result, the Mn^4+^ (0.54 Å) and Mn^2+^ (0.83 Å) ions are incorporated into the 16d Ti^4+^ (0.60 Å) and 8a Li^+^ (0.76 Å) sites, respectively, consistent with the lattice contraction of LMTO-O_2_ and the lattice expansion of LMTO-Ar, respectively.

### Phase-structure evolution

**Fig. 3:**
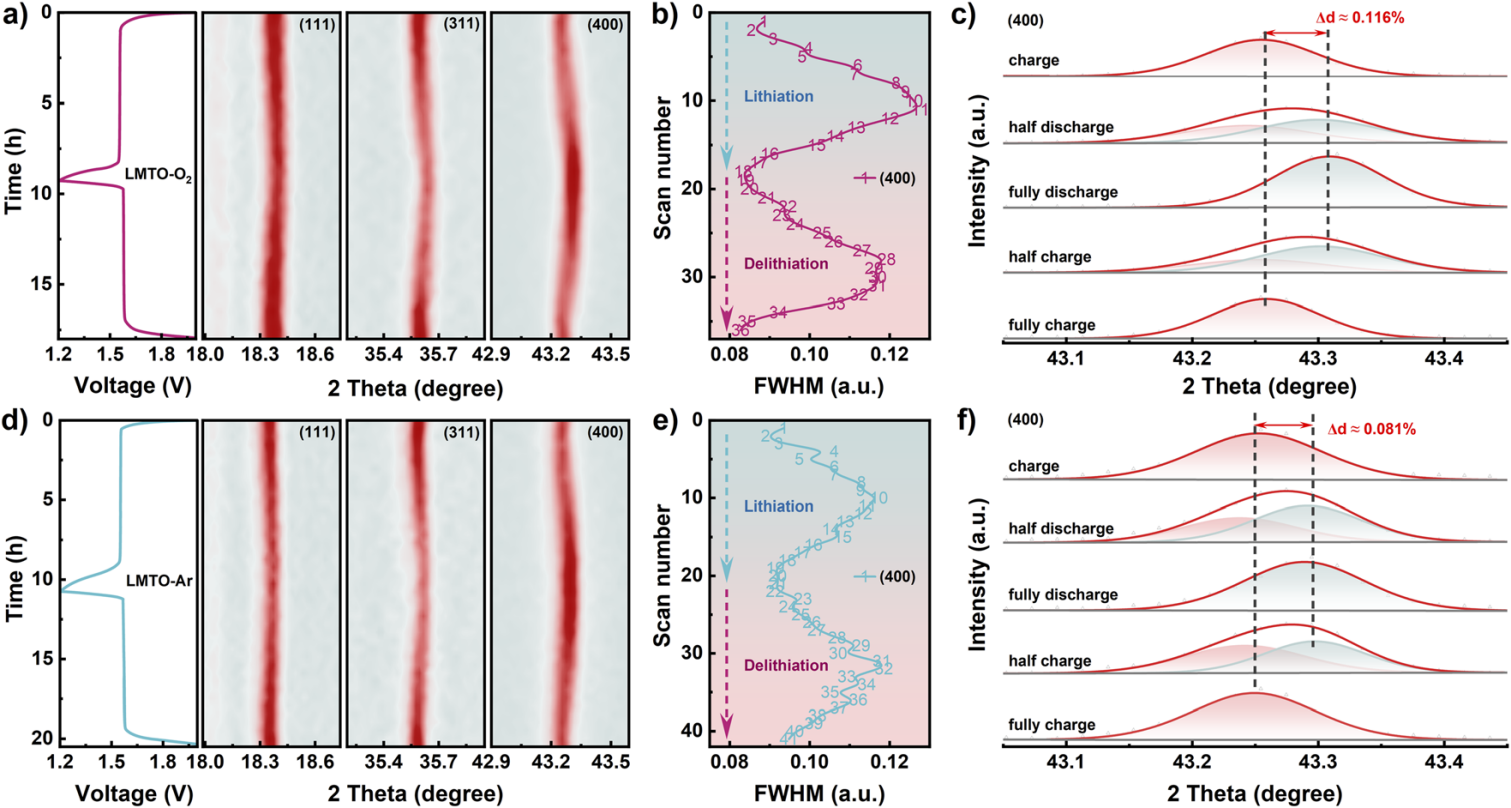
Galvanostatic discharge/charge curves and the corresponding *in situ* XRD patterns of (a) LMTO-O_2_ and (d) LMTO-Ar during cycling in the voltage window of 1.2–2.0 V at 0.1C. (b and e) The FWHM and (c and f) selected diffraction pattern of the (400) peak retrieved from *in situ* XRD patterns.

The structure evolution is evaluated by *in situ* XRD for LMTO-O_2_ and LMTO-Ar electrodes during galvanostatic cycling in the voltage window of 1.2–2 V at 0.1C. The discharge–charge curve, XRD patterns and corresponding contour map are shown in Fig. 3a, d, S7 and S8. Owing to the near-zero strain characteristic of LTO, the charged and discharged phases exhibit remarkably close positions of XRD peaks, making it very difficult to identify change in lattice parameters. Here, the (400) peak at a high angle is used for identifying phase evolution, of which the full width at half maximum (FWHM) is obtained through Lorentz fitting (Fig. 3b and e). It undergoes a non-monotonic change during the discharge process: gradually broadening in the beginning, reaching a maximum at half discharge and sharpening back at full discharge, and then a similar change is observed for the charge process. That is, the XRD patterns can be recognized as two peaks varying in intensity rather than one peak varying in position, as shown in Fig. S7 and S8. Accordingly, both LMTO-O_2_ and LMTO-Ar electrodes undergo a two-phase transition instead of a solid–solution reaction. To exhibit the detailed structural evolution in LMTO-O_2_ and LMTO-Ar during the discharge–charge process, we selected the XRD patterns of pristine, half discharge, fully discharge, half charge and fully charge states for comparison, as shown in Fig. 3c and f. In the charge state, there is only a (400) peak of spinel Li_4_Ti_5_O_12_. In the half discharge state, a new peak emerges on the right side of the (400) peak, indicating the phase transition from the spinel-structure (*α* phase) to the rock-salt structure (*β* phase). In the fully discharge state, the peak of the *α* phase is barely discernible, which is completely replaced by the new peak of the *β* phase. The lattice parameters of the *α* phase and *β* phase are assessed using the interplanar spacing (*d*-spacing) calculated from Bragg's formula, and the *d*-spacing change during the phase transition is larger for LMTO-O_2_ (Δ*d* ≈ 0.116%) than that for LMTO-Ar (Δ*d* ≈ 0.081%), owing to the different states of doped Mn ions under the two atmospheres.

### Switching the electrochemical oscillation

**Fig. 4:**
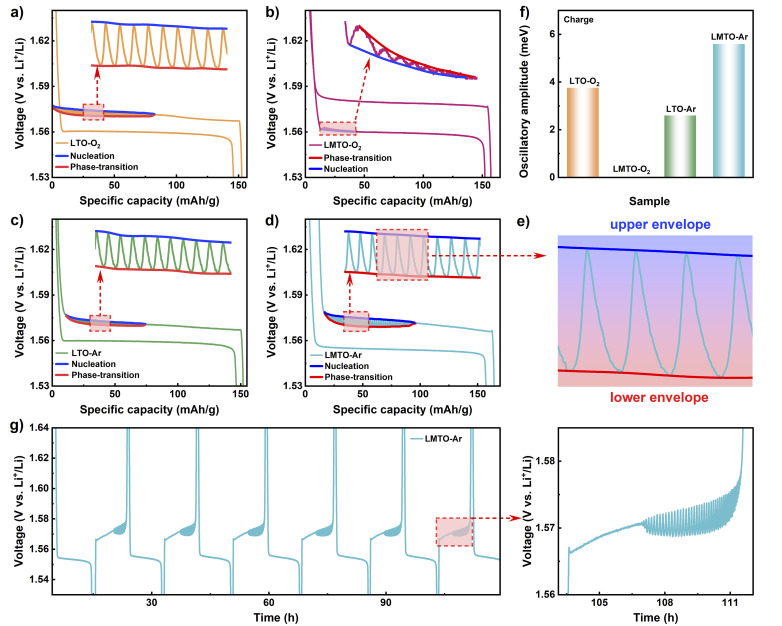
(a–d) Galvanostatic charge/discharge curves at 0.1C and the enlarged view in the oscillation region of LTO-O_2_, LMTO-O_2_, LTO-Ar and LMTO-Ar, respectively. The peak and valley of periodic fluctuation are delineated by blue and red lines. (e) In the oscillatory regime, a comparison of maximum oscillatory amplitude of the four samples. (f) The enlarged view in the oscillation region of LMTO-Ar. (g) The voltage–time curve and magnified view of LMTO-Ar at 0.1C.

Fig. 4a–d shows the charge–discharge curves and the enlarged view in the oscillatory region of LTO-O_2_, LTO-Ar, LMTO-O_2_ and LMTO-Ar at 0.1C. For LTO-O_2_ and LTO-Ar, the voltage oscillation appears only in the back of the charge voltage plateau, which is analogous to the oscillatory phenomenon documented by Li *et al.*^36^ In comparison, LMTO-Ar exhibits enhanced amplitude of charge oscillation, and the oscillation of LMTO-O_2_ emerges at the onset of discharge (Fig. 4e), which is the first observation of oscillation phenomenon within the initial region of discharge plateau. And the voltage oscillation of LMTO-O_2_ and LMTO-Ar can persist in subsequent cycles (Fig. 4g and S9). As mentioned above, the peak and valley envelopes within the oscillatory region correspond to the overpotentials of nucleation and phase-transition steps, respectively, and their separation is attributed to the hysteretic phase transition after the nucleation. Additionally, the asymmetric peak and valley in each oscillation period reflect the instantaneous nucleation and subsequent gradual phase transition, as shown in Fig. 4f.

Furthermore, we sintered the LMTO in O_2_ and Ar atmospheres alternatively to obtain the samples as (i) LMTO-Ar, LMTO-Ar → O_2_, and LMTO-Ar → O_2_ → Ar and (ii) LMTO-O_2_, LMTO-O_2_ → Ar, and LMTO-O_2_ → Ar → O_2_. Fig. S10 and S11 show optical images, the galvanostatic charge/discharge curves, and the UV-vis DRS results. The reversible color transformation (bright pink for O_2_*vs.* blush white for Ar) and a characteristic absorption peak of Mn^4+^ manifest that the annealing atmosphere successfully altered the valence state of Mn ions. Compared with Ar-annealed LMTO (LMTO-Ar, LMTO-O_2_ → Ar and LMTO-Ar → O_2_ → Ar) samples, the O_2_-annealed LMTO (LMTO-O_2_, LMTO-Ar → O_2_ and LMTO-O_2_ → Ar → O_2_) samples elevate the lithiation/de-lithiation voltage plateaus for approximately 10 mV and lose the electrochemical oscillation during charging, suggesting that the Mn-ion state is reversibly modulated by altering the annealing atmosphere. To track the transformation process of Mn ions, the LMTO-O_2_ samples were subjected to an annealing process under an Ar atmosphere at 400, 600 and 800 °C, respectively. As shown in Fig. S12, the voltage plateau lowers and the amplitude strengthens gradually on increasing the annealing temperature, which may be attributed to the reduction of Mn ions and subsequent migration (Ti_16d_ to Li_8a_). Conversely, the electrochemical properties of LMTO-Ar are gradually changed into those of LMTO-O_2_ through annealing under a O_2_ atmosphere, as shown in Fig. S13. Thereby, the Mn^4+^ ions at 16d sites can induce the oscillatory phenomenon at the initial discharge plateau, and the Mn^2+^ ions at 8a sites can enhance the oscillatory phenomenon in the back of the charge plateau.

At the onset of phase-separation region, a damped voltage oscillation is observed for the LMTO-O_2_ samples. In order to reproduce this phenomenon, periodic rest steps are inserted into the discharge and charge processes, that is, a 2 h rest is applied to interrupt the galvanostatic (dis)charging at 0.1C for each 1.5 h, which makes the electrode relax to the equilibrium state and then restarts the electrochemical reaction, as shown in Fig. S14. Here, re-applying the current after each relaxation interval produces voltage overshooting, which is due to the massive instantaneous nucleation of active particles. After the rest during discharging, LMTO-O_2_ exhibits an evident damped voltage oscillation, whereas no oscillation is observed for LMTO-Ar, which is further verified by the LMTO-Ar → O_2_, LMTO-Ar → O_2_ → Ar, LMTO-O_2_ → Ar and LMTO-O_2_ → Ar → O_2_ samples, as shown in Fig. S14d and S14e. Thereby, the damped oscillation during discharging can be triggered by the doped Mn ions, of which the valence state and lattice site are switched by the sintering atmosphere.

Besides the electrochemical oscillation, the other electrochemical performances are also evaluated for LTO-O_2_, LTO-Ar, LMTO-O_2_ and LMTO-Ar. As shown in Fig. S15, LMTO-O_2_ exhibits an evidently lower reversible capacity at 10C than the other three samples with distinct charge oscillations, which might be owing to the LMTO-O_2_ sample possessing a larger lattice mismatch. Meanwhile, the LMTO-Ar sample delivers outstanding cycling stability, achieving an exceptional capacity retention of 93.7% after 3000 cycles at 5C, compared with 87.6% for LTO-Ar (Fig. S16). However, the LMTO-O_2_ sample exhibits a capacity retention of 82.9% after 3000 cycles at 5C, which is inferior to that of LTO-O_2_ (87.1%). Thereby, the doped LTO samples exhibit different electrochemical performances, such as rate and cycling performances. The CV measurements of the LMTO-Ar and LMTO-O_2_ electrodes were conducted at scan rates from 0.02 to 0.4 mV s^−1^ in a voltage range of 1.2–2 V (*vs.* Li^+^/Li), as shown in Fig. S17, and the peak current (*I*_*P*_) is linearly related to the square root of the scan rate (*v*^1/2^). According to the Randles–Sevcik equation,^37–39^ the Li^+^ diffusion kinetics is calculated for both samples, and LMTO-Ar shows smaller slope values than LMTO-O_2_ during the lithiation process, suggesting inferior reaction kinetics. Thus, LMTO-Ar presents better rate and cycle performances but inferior lithiation kinetics.

### Doped sites and electrochemical kinetics

**Fig. 5:**
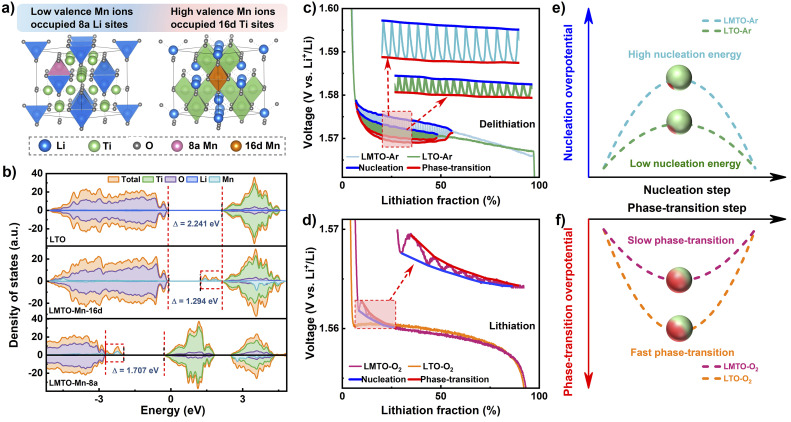
(a) Schematic illustration of LMTO-8a and LMTO-16d structures visualized using VESTA. (b) Density of states calculation of LTO, LMTO-16d and LMTO-8a. (c) Galvanostatic charge curves of LMTO-Ar and LTO-Ar samples at 0.1C. (d) Galvanostatic discharge curves of LMTO-O_2_ and LTO-O_2_ samples at 0.1C. (e) Schematic illustration of nucleation overpotential of LMTO-Ar and LTO-Ar samples during de-lithiation. (f) Schematic illustration of phase-transition overpotential of LMTO-O_2_ and LTO-O_2_ samples during lithiation.

As shown in Fig. 5a, the structure of Mn atoms at octahedral and tetrahedral sites is visualized using VESTA, and DFT calculations are conducted to probe the changes in the band structure and coordination environment resulting from Mn substitution at the 8a and 16d sites in spinel LTO.^27,40^ The total densities of states (TDOS) of LMTO-O_2_ (Mn occupied on 16d) indicate that additional energy states below the conduction band reduce the band gap (from 2.241 eV to 1.294 eV), thereby enhancing the electronic conductivity of the host. The projected density of states (PDOS) shows that the additional electronic states mainly originate from the hybridization between Mn 3d and O 2p orbitals (Fig. S18). This hybridization introduces an electron-buffering band, which enhances the electronic polarizability of the LTO lattice and provides an extra electronic compensation pathway during Li^+^ insertion and extraction. Additionally, the hybridization between Mn 3d and O 2p orbitals filled at the top of the valence band for the LMTO-Ar with Mn substitution in Li (8a) sites, resulting in a band gap reduction of about 0.53 eV (Fig. 5b and S19). Interestingly, the strong local binding between Mn 3d and O 2p orbitals causes the TDOS to shift to lower energy,^21,41^ and the strengthened Mn–O interactions exert a pinning effect on the framework and harden the diffusion channels. Besides, the substitution of Li^+^ by Mn^2+^ at the 8a site hinders Li^+^ transport and consequently elevates the nucleation barrier, because the two-phase transition from spinel Li_4_Ti_5_O_12_ to rock-salt Li_7_Ti_5_O_12_ is accomplished by Li^+^ ion migration from tetrahedral 8a sites to octahedral 16c sites.^42^ Therefore, both the electronic structure and Li-ion pathway have been reconfigured for LMTO-O_2_ and LMTO-Ar, compared with the pristine LTO.

The voltage curves between doped and pristine samples were compared to analyse the impact of the coordination environment on the nucleation and phase-transition kinetics. As shown in Fig. 5c, LMTO-Ar exhibits a lower phase-transition envelope and a higher nucleation envelope compared with LTO-Ar, indicating a higher nucleation energy barrier, as shown in Fig. 5e, possibly owing to that the Mn ions at 8a Li sites hinder the Li^+^ insertion/extraction. Fig. 5d shows reduced overpotentials for nucleation and phase transition at the onset of lithiation for LMTO-O_2_, which may be attributed to the enhanced electronic conductivity resulting from Mn ions at the 16d Ti sites. The oscillation period evidently reduces by increasing the overpotential, indicating that the phase transition of a single particle is accelerated by the higher overpotential, as shown in Fig. 5f. In other words, the slow phase-transition step makes the electrochemical oscillation clear.

### Numerical simulation

**Fig. 6:**
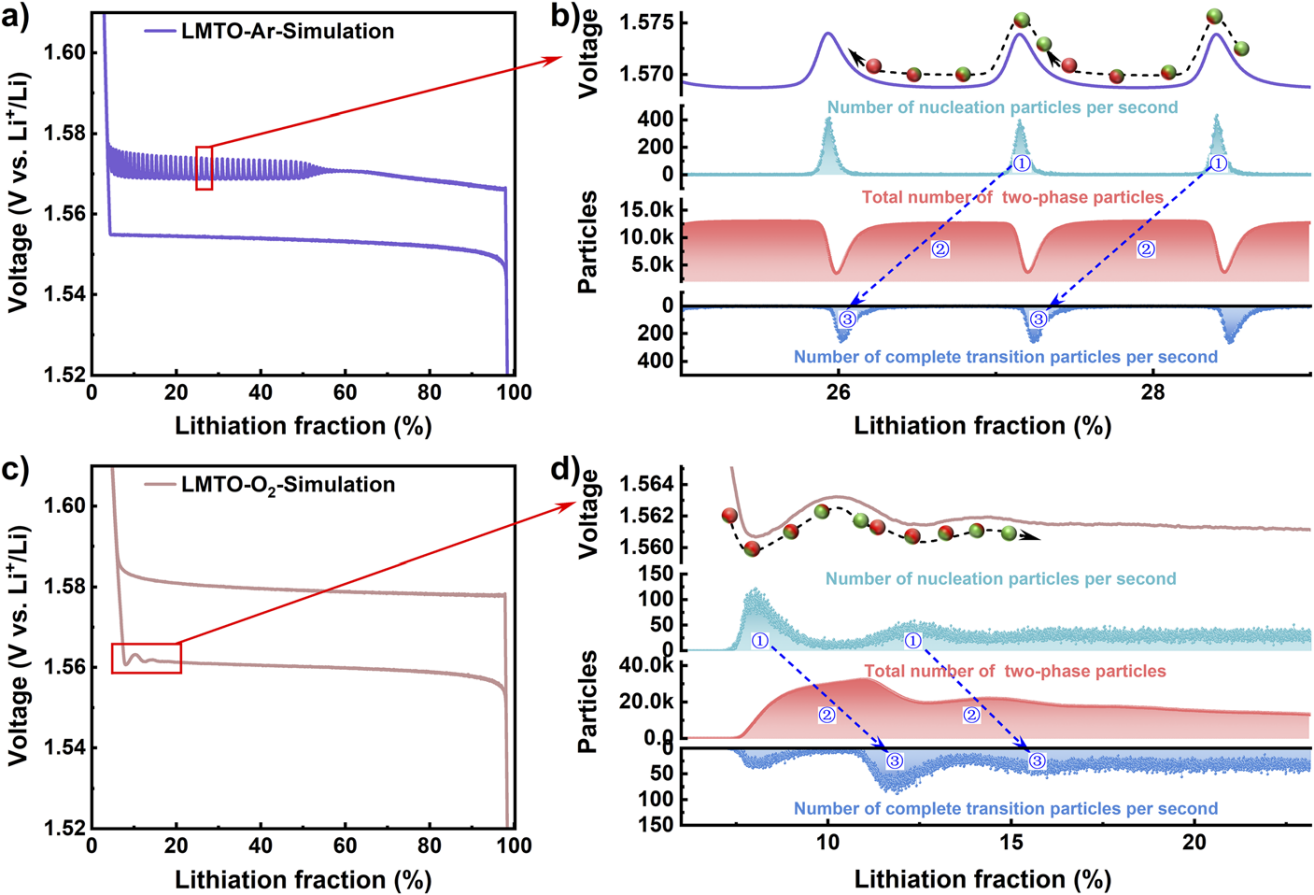
The simulated voltage profiles at 0.1C of LMTO-Ar (a) and LMTO-O_2_ (c). (b and d) The schematic presentation of phase transition along with the electrochemical oscillation and simulated particle population responding as fluctuation of the lithiation degree, including nucleation particles per second, total two-phase particles, and completed-transition particles per second.

The electrochemical oscillations are simulated using a model of a million-particle electrode with proper nucleation and phase-transition kinetics, both of which are described by the Butler–Volmer equation as eqn (a) and (b).a
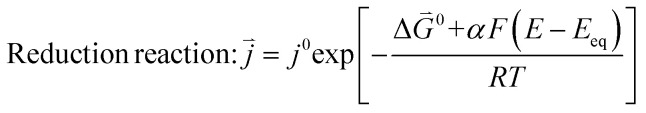
b
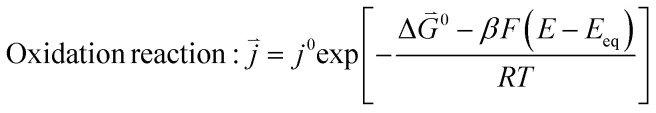
where 
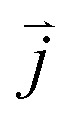
 is the total reduction rate, 
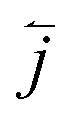
 is the total oxidation rate, *j*^0^ is the exchange current density, *E* is electrode potential, *E*_eq_ is equilibrium potential for a two-phase transition, 
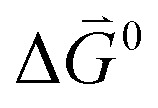
 and 
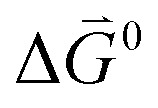
 are reduction and oxidation activation energies, respectively, *F* is the Faraday constant, *R* is the ideal gas constant, *T* is absolute temperature, and *α* and *β* are the transfer coefficients of reduction and oxidation reactions, respectively. *α* and *β* are set as ∼4 for the phase-transition step and ∼28 for the nucleation step, because the former is normally a multi-electron reaction and the latter is usually a many-electron reaction, and the other parameters of each particle are set properly within a normal distribution, in accordance with the real electrode. Through numerical simulations, both the galvanostatic discharge and charge curves, as well as the reaction details, are achieved excellently for LMTO-O_2_ and LMTO-Ar, as shown in Fig. 6. The electrochemical reaction in each oscillation period can be deconstructed into three steps: (1) the massive nucleation at a high overpotential makes the population of active particles increase dramatically, (2) the phase transition of abundant active particles corresponds to a low overpotential, and (3) many active particles complete the phase transition, resulting in elevated overpotential. Therefore, the combination of a high nucleation overpotential and a long lifetime of active particles (*i.e.*, the phase-transition step lags far behind the nucleation step at a high overpotential) provides a unique cradle for the emergence of electrochemical oscillation.

## Conclusions

In this work, we developed a valence-engineering strategy to trigger the electrochemical oscillation of the Mn-doped LTO, which is enhanced in the back of the charge plateau for the LMTO-Ar sintered under an Ar atmosphere and damped in the beginning of discharging for the LMTO-O_2_ sintered under an O_2_ atmosphere. Owing to different redox conditions, the Mn^4+^ and Mn^2+^ ions are incorporated into the 16d Ti^4+^ site for LMTO-O_2_ and 8a Li^+^ site for LMTO-Ar, respectively, resulting in different nucleation and phase-transition kinetics between the two samples. Combining with the simulation of a million-particle electrode, the electrochemical oscillation can be interpreted using the split overpotentials of nucleation and phase-transition steps, owing to the high overpotential of the nucleation step and the hysteresis of the phase-transition step. Thereby, this investigation provides a viable strategy for regulating electrochemical oscillation through ion doping and proposes an innovation perspective on understanding electrochemical oscillation using the electrode kinetics.

## Author contributions

W. T. Ji, D. Li and H. S. Zhou conceived and designed the research. D. Li, Y. Chen, and H. S. Zhou supervised the research. W. T. Ji and Y. Q. Huang performed the experiments and characterization of materials. J. Bai and D. Li conducted DFT calculations and numerical simulation. W. T. Ji and J. Bai wrote the original draft. All the authors commented on and revised the paper.

## Conflicts of interest

There are no conflicts to declare.

## Supplementary Material

SC-OLF-D6SC05042E-s001

## Data Availability

The authors have cited additional references within the supplementary information (SI).^43–51^ Supplementary information: general procedures, syntheses, characterization, properties, and a comparison of electrochemical oscillation phenomenon are shown in Fig. S1−S19 and Tables S1−S4 (PDF). See DOI: https://doi.org/10.1039/d6sc05042e.
