# Supplementary material for: Electrochemical oscillation triggered by the doped Mn ions in spinel Li4Ti5O12
Source: Chem Sci. 2026 Jul 21;17(38):18152–60. doi: 10.1039/d6sc05042e (PMC13462858; doi:10.1039/d6sc05042e)
Supplement: Supplementary file 1 [file SC-017-D6SC05042E-s001.pdf]

Supplementary Information (SI) for Chemical Science.
This journal is © The Royal Society of Chemistry 2026

Supporting Information for

Electrochemical Oscillation Triggered by the Doped Mn Ions in Spinel $Li_4Ti_5O_{12}$

Wenting Ji,†[a] Jie Bai,†[c] Yuqin Huang,[a] De Li,*[a] Yong Chen*[b] and Haoshen Zhou[d]

[a] State Key Lab of Tropic Ocean Engineering Materials and Materials Evaluation, School of Materials Science and Engineering, Hainan Provincial Key Laboratory of Research on Utilization of Si−Zr−Ti Resource, Hainan University, Haikou 570228, China.
[b] School of Materials and Energy, Foshan University, Foshan 528000, China.
[c] Department of Applied Chemistry, School of Chemistry and Chemical Engineering, Chongqing University, Chongqing 401331, China.
[d] Center of Energy Storage Materials & Technology, College of Engineering and Applied Sciences, Jiangsu Key Laboratory of Artificial Functional Materials, National Laboratory of Solid-State Microstructures and Collaborative Innovation Center of Advanced Microstructures, Nanjing University, Nanjing 210093, China.
† Wenting Ji and Jie Bai contributed equally to this work.
*E-mail: lidehainu@hainanu.edu.cn (D. Li); ychen2002@163.com (Y. Chen)

Number of pages: 12
Number of Figures: 19
Number of Tables: 4

Experimental section

Synthesis Procedures

$Li_4Ti_5O_{12}$ (LTO) and Mn-doped LTO materials are synthesized by a simple spray drying and high-temperature sintering method[43] with $LiOH{\cdot}H_2O$ (Macklin, AR 98%), $C_{16}H_{36}O_4Ti$ (Aladdin, 99.0%), and $MnCO_3$ (Aladdin, 99.99%) as raw materials. Mn-doped LTO precursor is prepared with a 3.8: 0.05: 5 molar ratio of $LiOH{\cdot}H_2O$, $MnCO_3$ and $C_{16}H_{36}O_4Ti$. The precursor powder is sintered at 800 °C for 10 h in O_2 or Ar to obtain the LMTO-O_2 or LMTO-Ar, respectively. For comparison, the undoped LTO (3.8: 5 molar ratio of $LiOH{\cdot}H_2O$ and $C_{16}H_{36}O_4Ti$) are synthesized using the same method, namely, LTO-O_2 and LTO-Ar.

Characterizations of materials

The X-ray powder diffraction (XRD) patterns are collected with a Bruker D2 Advance diffractometer, using Cu Ka radiation. Rietveld refinement program GSAS-II is utilized to analyse the XRD data. The Raman spectra are collected by a Raman spectrometer (Raman, Thermo Scientific DXR xi) with a laser at a wavelength of 532 nm. Electron Paramagnetic Resonance (EPR, Bruker A300-10/12) spectroscopy is performed at 77 K to identify the valence states of Mn-doped LTO. Ultraviolet-Visible diffuse reflectance spectra (UV-vis DRS) are acquired on the Model Hitachi UH5700 spectrophotometer using the $CaSO_4$ as the reference. The morphology and structure of samples are characterized by a scanning electron microscope (SEM, Verios G4 UC, Thermo) and a transmission electron microscope (TEM, FEI Talos F200s) with Energy-Dispersive X-ray (EDX) detector. X-ray photoelectron spectroscopy (XPS) is conducted by using a Thermofisher ESCALAB 250xi spectrometer with aluminium as the X-ray source.

Measurements of batteries

Electrochemical performances are evaluated by using 2032-type half-cells. The 42.5 wt% active material, 15 wt% polytetrafluoroethylene (PTFE) and 42.5 wt% acetylene black are homogeneously mixed into a paste and rolled into a uniform thin film. Then, this film with a mass loading of *ca.* 2 $mg{\cdot}cm^{-2}$ is punched into small pieces and pressed onto the carbon paper as the working electrode. The half cells are assembled in an argon-filled glove-box with Li metal as the counter electrode, Celgard 2500 separator and 1M LiTFSI electrolyte (ethyl carbonate (EC): diethyl carbonate (DEC) with a volume ratio of 1:1). The galvanostatic charge and discharge measurements are performed on Hokuto Denko battery test system, the cyclic voltammetry (CV) and electrochemical impedance spectroscopy (EIS) measurements are conducted on Biologic workstation (VSP-300).

In-situ X-ray powder diffraction

As described in our previous work,[44] a Φ14 mm hole is drilled on the bottom case of a normal 2032 coin cell, on which a 0.2 mm thick Be sheet as an X-ray window is firmly stuck with a thermoplastic film, and a Φ10 mm thin electrode film on a Ti mesh is intimately attached to the window. The in-situ XRD measurement is conducted by using a Bruker D2 Advance diffractometer with Cu Ka radiation, and the XRD patterns are collected every 30 minutes, which are calibrated by using the diffraction peak of beryllium (Be). Simultaneously, the galvanostatic charge/discharge measurements are conducted on Biologic workstation within a voltage range of 1.2-2 V at 0.1 C.

Computational details

All density functional theory (DFT) calculations are carried out using the Vienna Ab initio Simulation Package (VASP),[45,46] with DFT + U corrections applied to the Mn.[47] The Kohn-Sham equations are solved using the projector augmented-wave (PAW) approach,[48,49] and the exchange-correlation potential is described within the generalized gradient approximation (GGA) constructed by Perdew, Burke and Ernzerhof.[50,51] In this work, geometry optimization is performed using a 1a × 3b × 1c supercell, and convergence is considered achieved when the force on each atom is less than 0.01 eV $Å^{-1}$, and the energy convergence criterion is set to 10^{-6} eV. For all calculations, the plane-wave cutoff energy is set to 600 eV. The Brillouin zone integrations are performed with 5 × 2 × 5 and 10 × 4 × 10 Γ-centered Monkhorst-Pack k-point meshes for geometry optimization and density of states calculations, respectively.

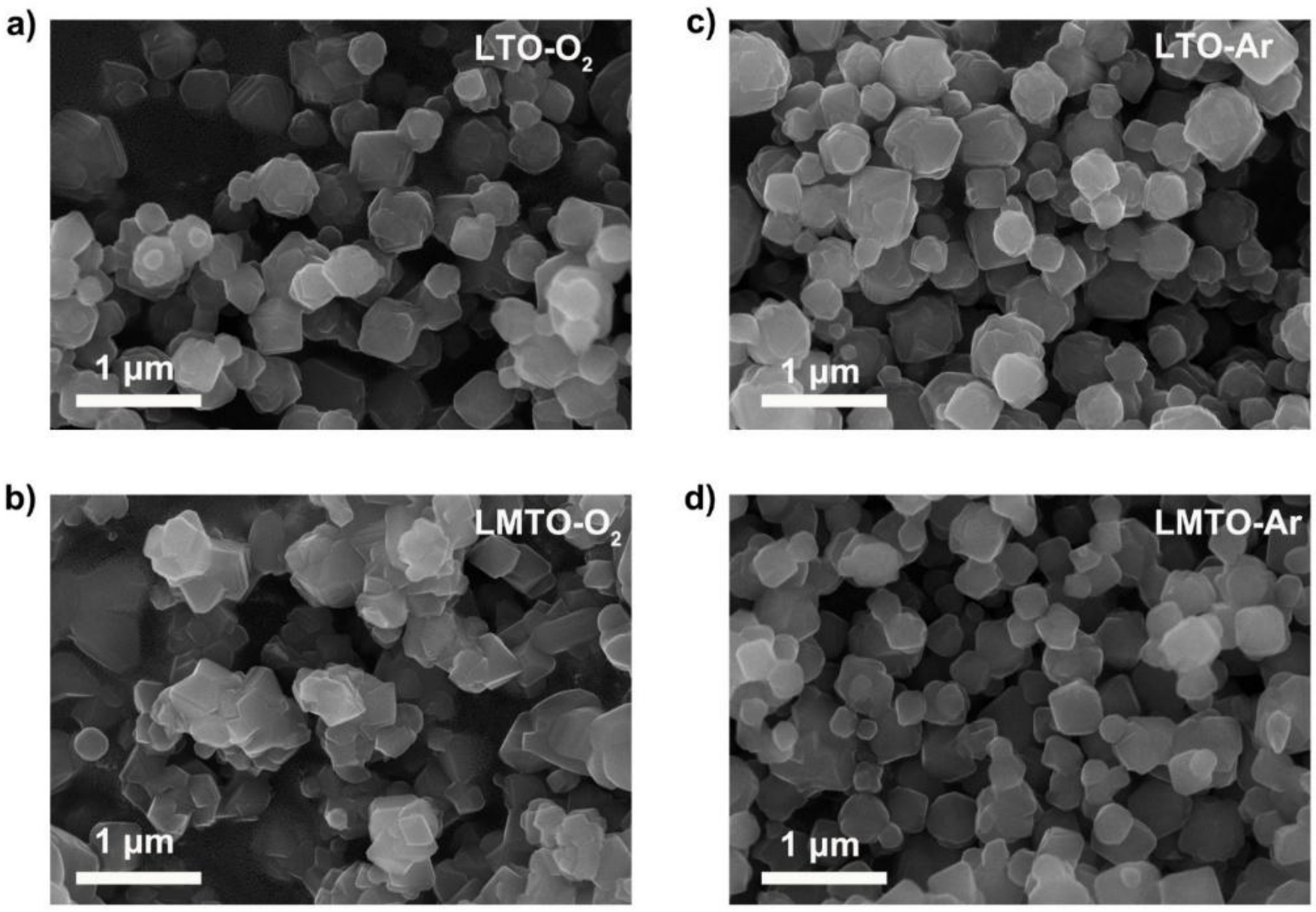

Fig. S1. The SEM images of (a) LTO-O_2, (b) LMTO-O_2, (c) LTO-Ar and (d) LMTO-Ar.

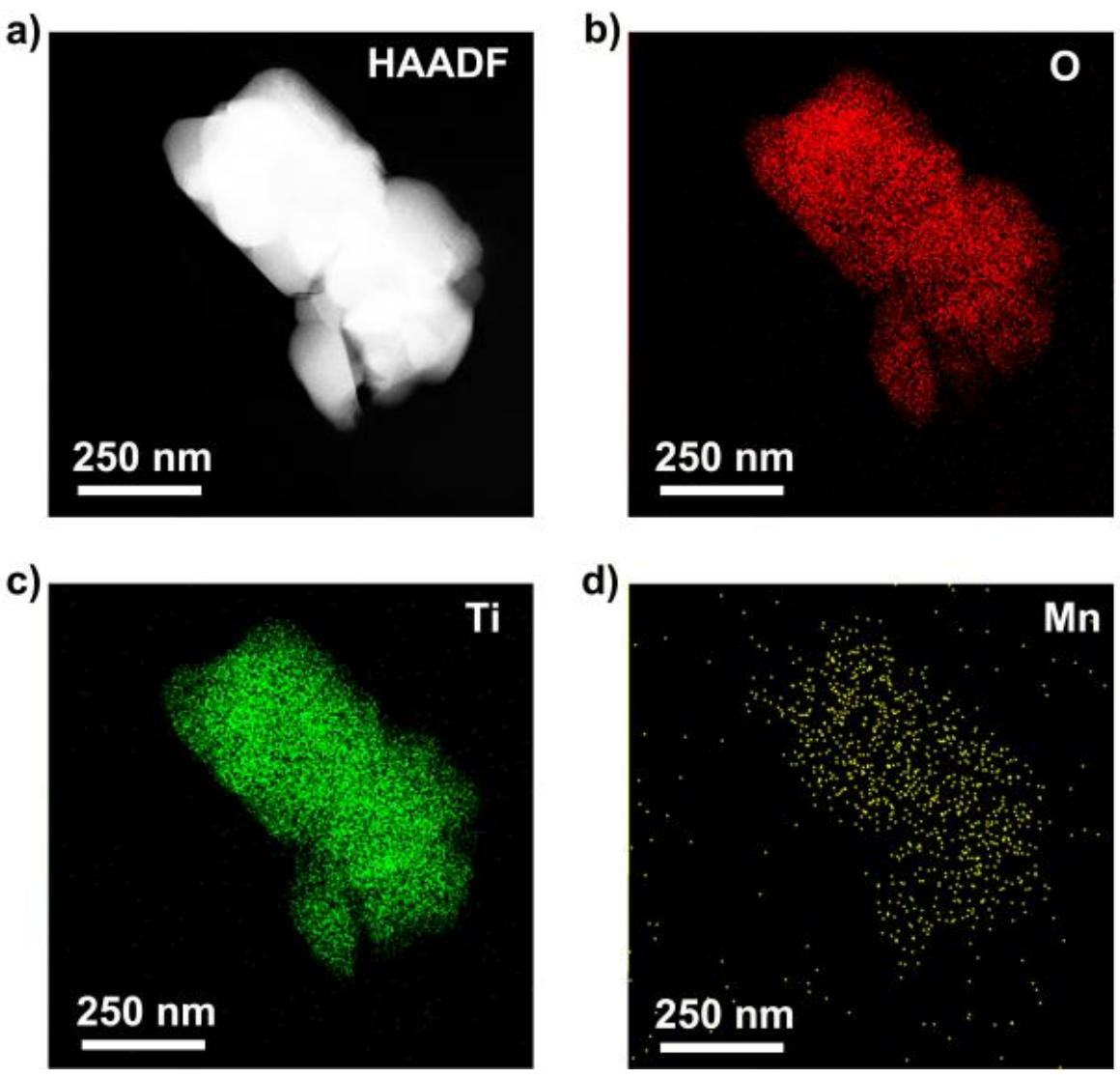

Fig. S2. (a) High-angle annular dark field image and (b-d) the relevant element mapping images of LMTO-O_2 particles.

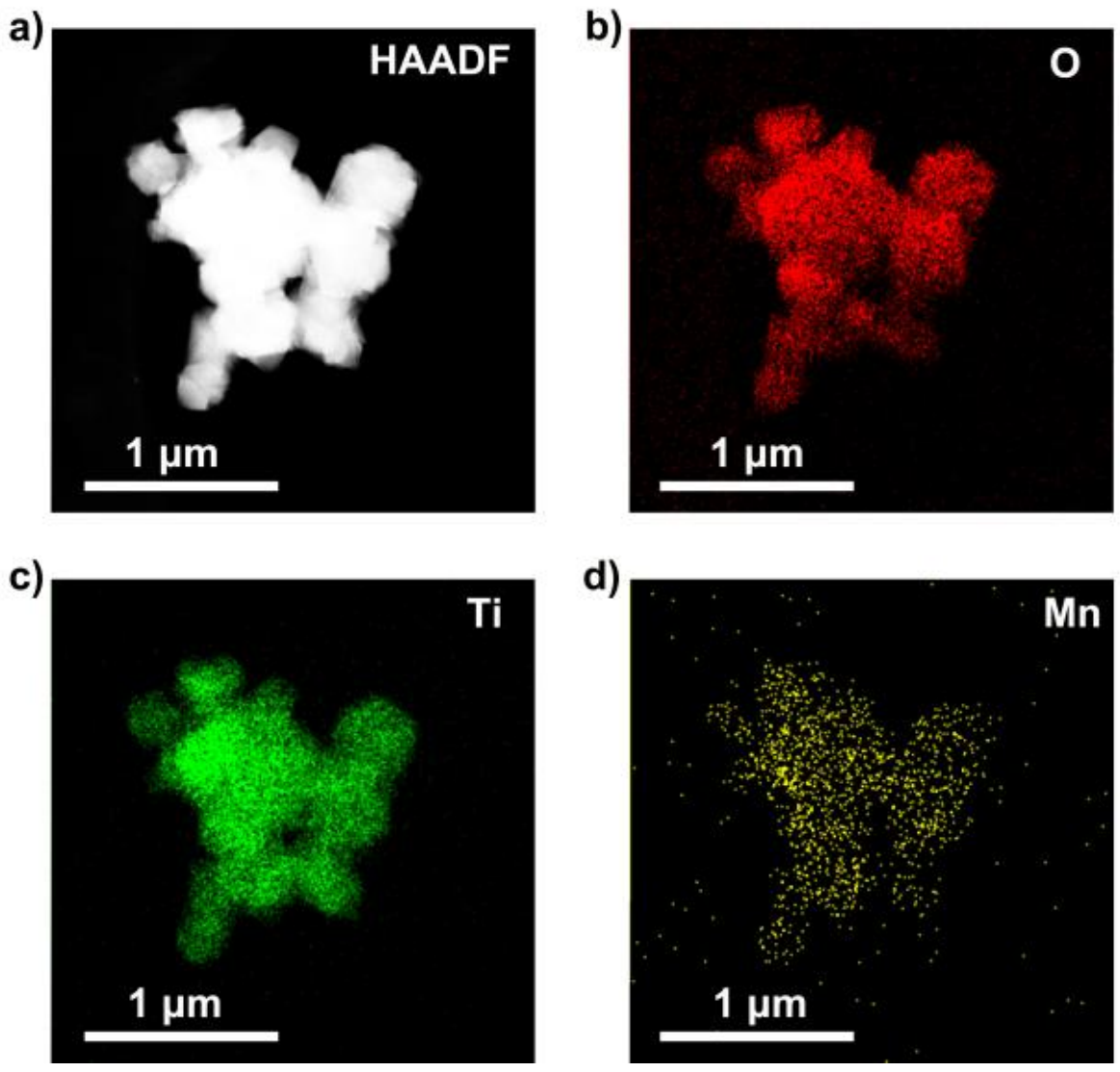

Fig. S3. (a) High-angle annular dark field images and (b-d) the relevant element mapping images of LMTO-Ar particles.

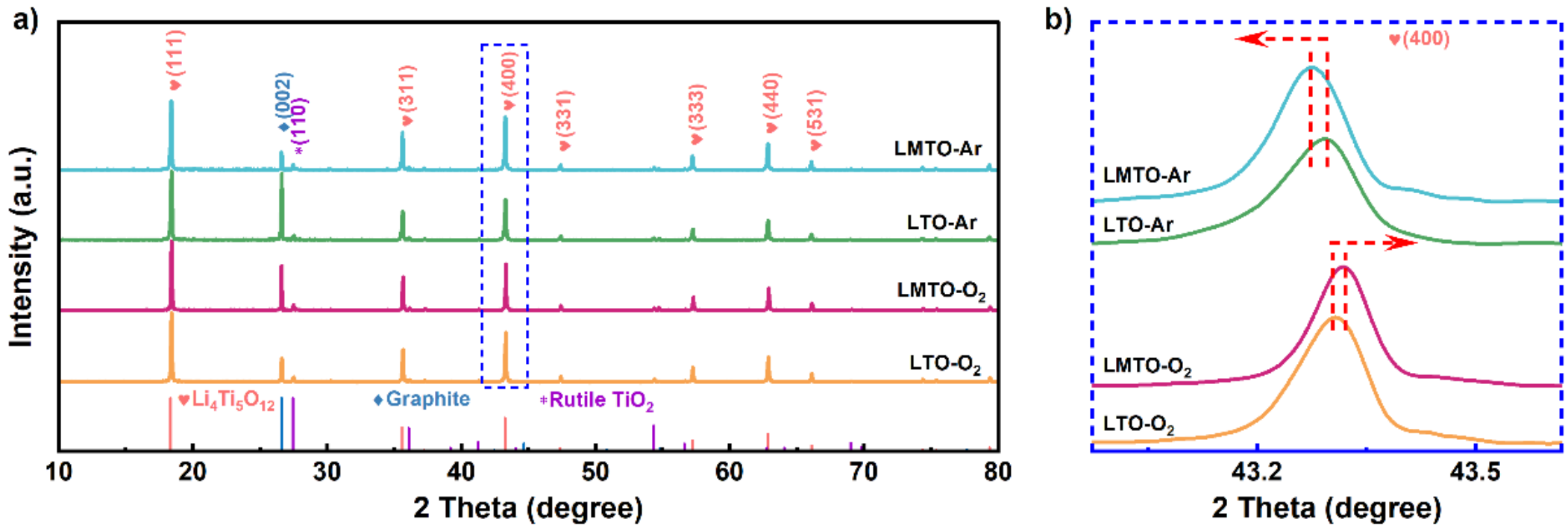

Fig. S4. (a) The XRD patterns of LTO-O_2, LMTO-O_2, LTO-Ar and LMTO-Ar, (b) the magnification region of (400) peak.

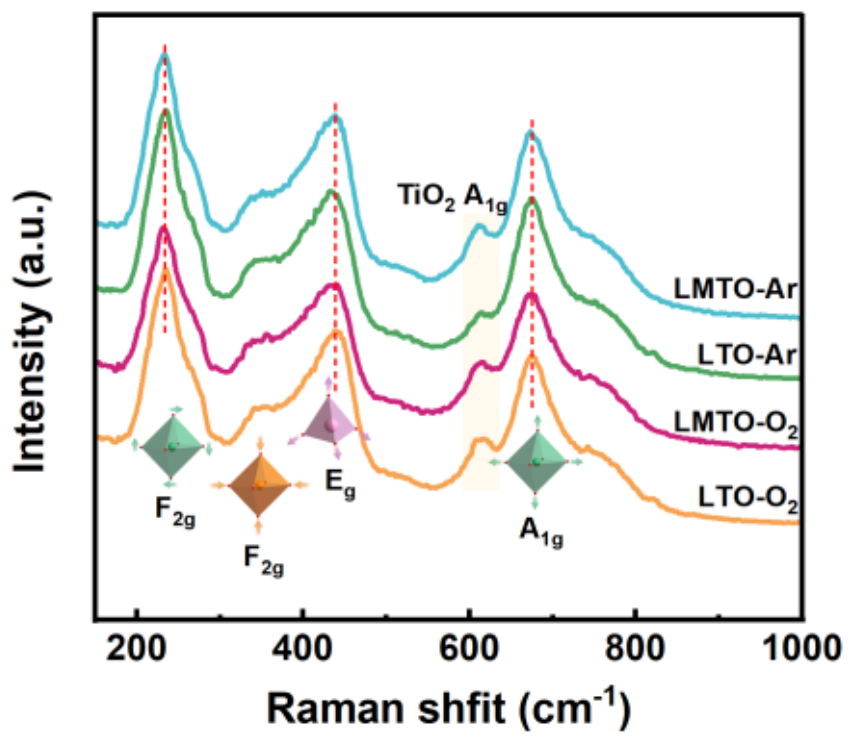

Fig. S5. The Raman spectra of LTO-O_2, LMTO-O_2, LTO-Ar and LMTO-Ar.

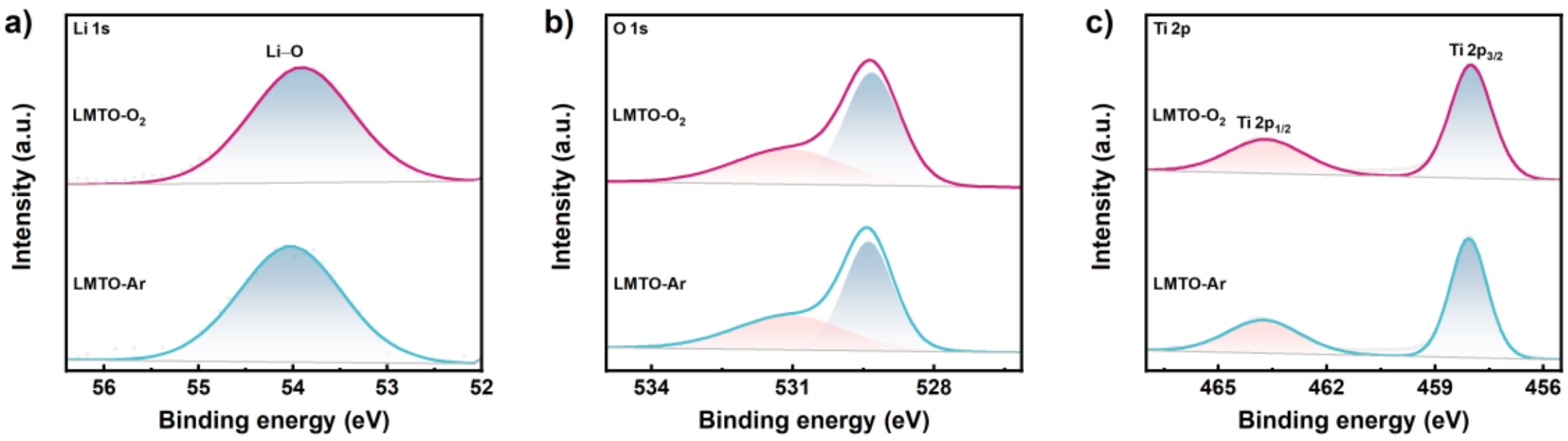

Fig. S6. The XPS spectra of (a) Li 1s, (b) O 1s and (c) Ti 2p of LMTO-O_2 and LMTO-Ar.

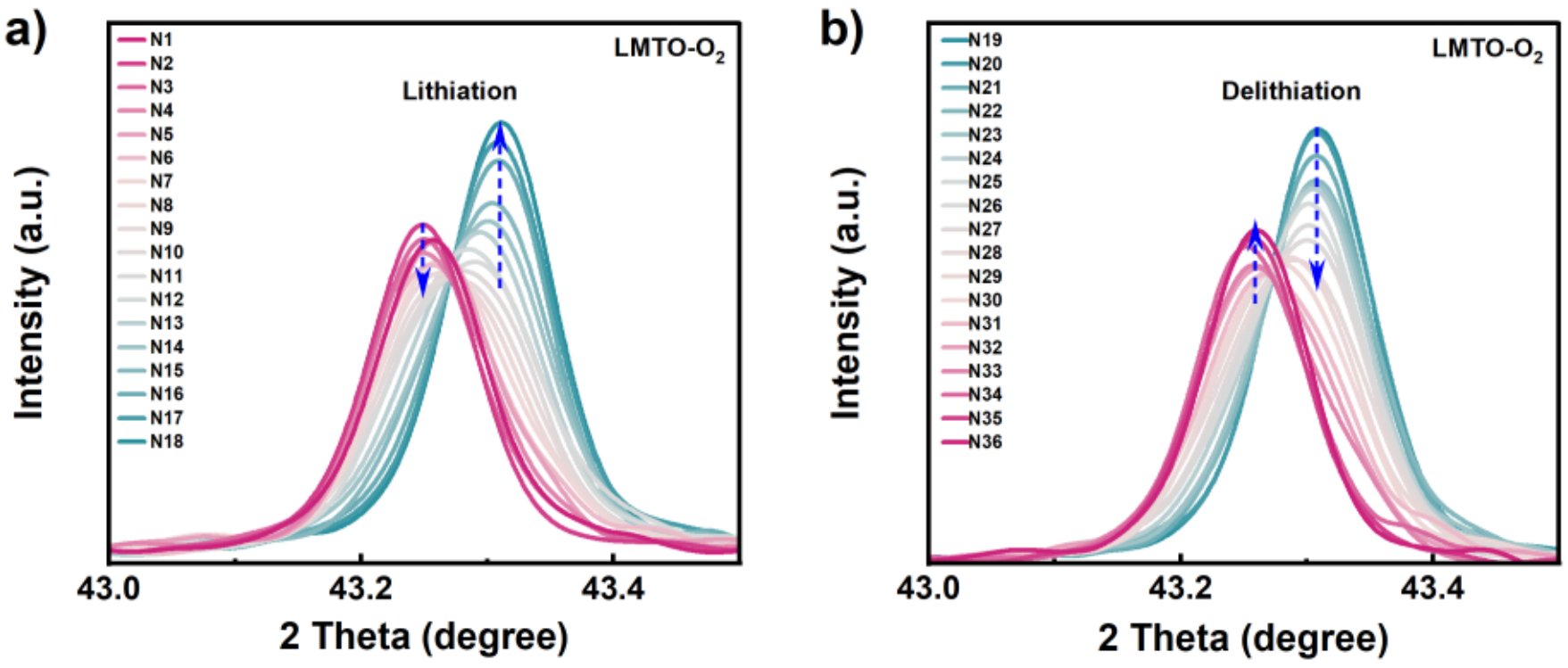

Fig. S7. Selective region of the in-situ XRD patterns from 43.0° to 43.5° for the LMTO-O_2 during (a) lithiation and (b) de-lithiation at 0.1 C.

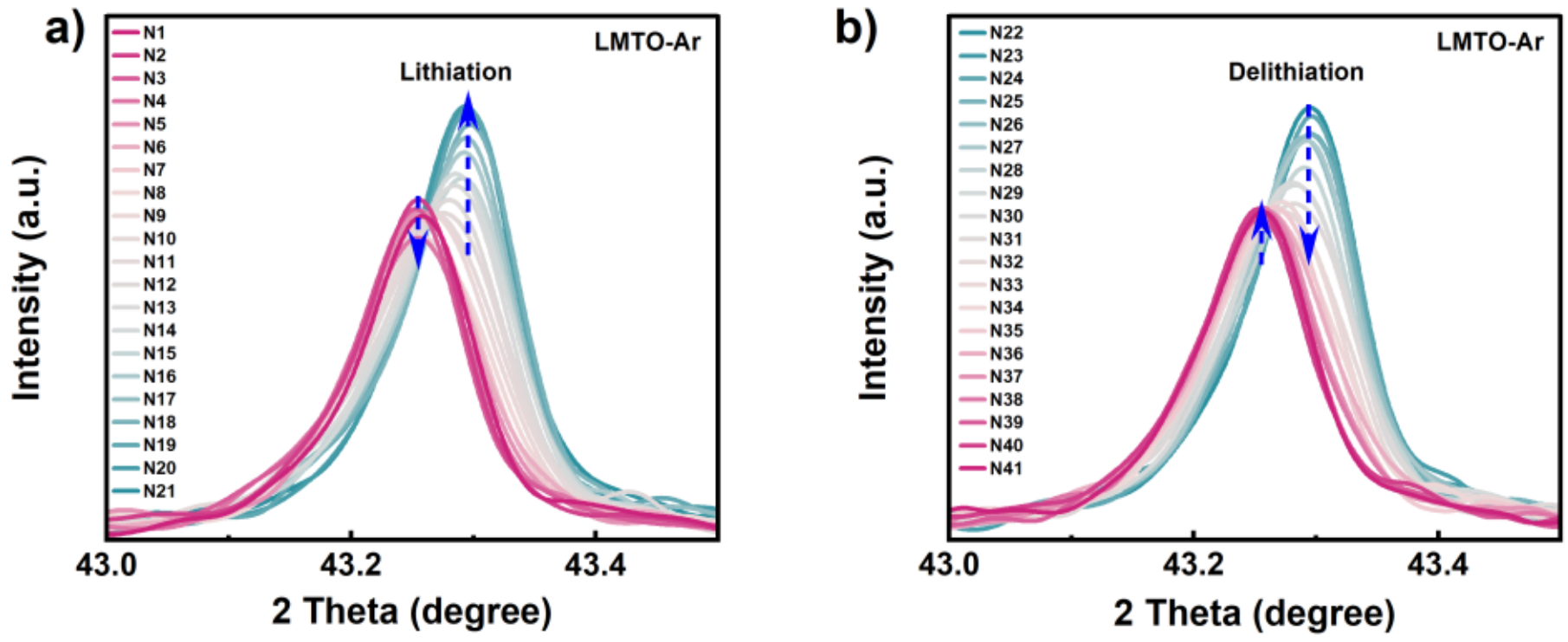

Fig. S8. Selective region of the in-situ XRD patterns from 43.0° to 43.5° for the LMTO-Ar during (a) lithiation and (b) de-lithiation at 0.1 C.

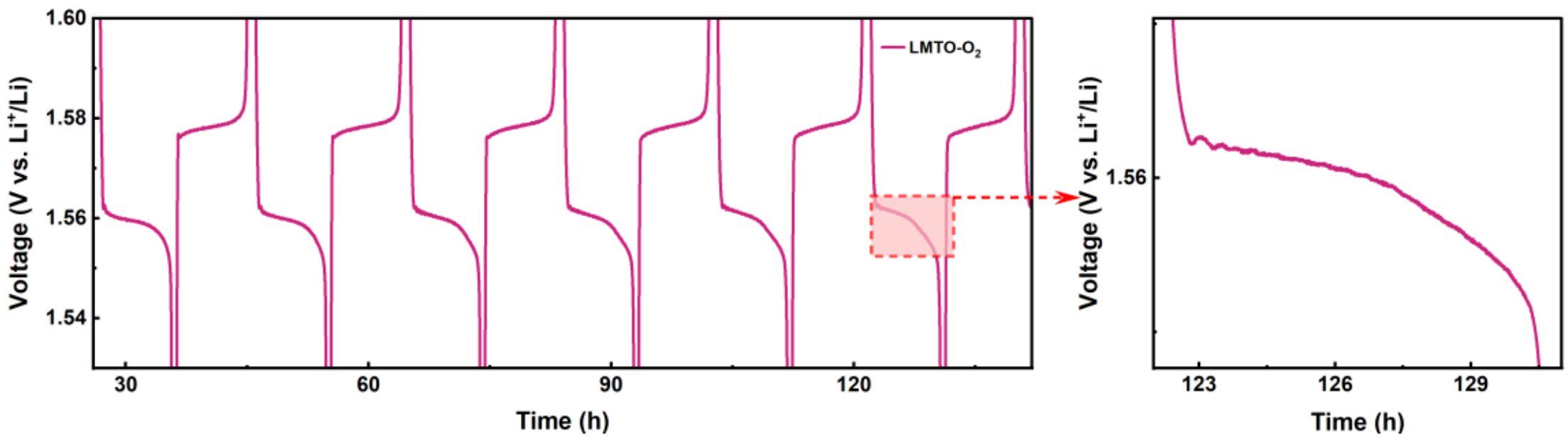

Fig. S9 Galvanostatic charge/discharge curves and the enlarged views of LMTO-O_2 at 0.1C.

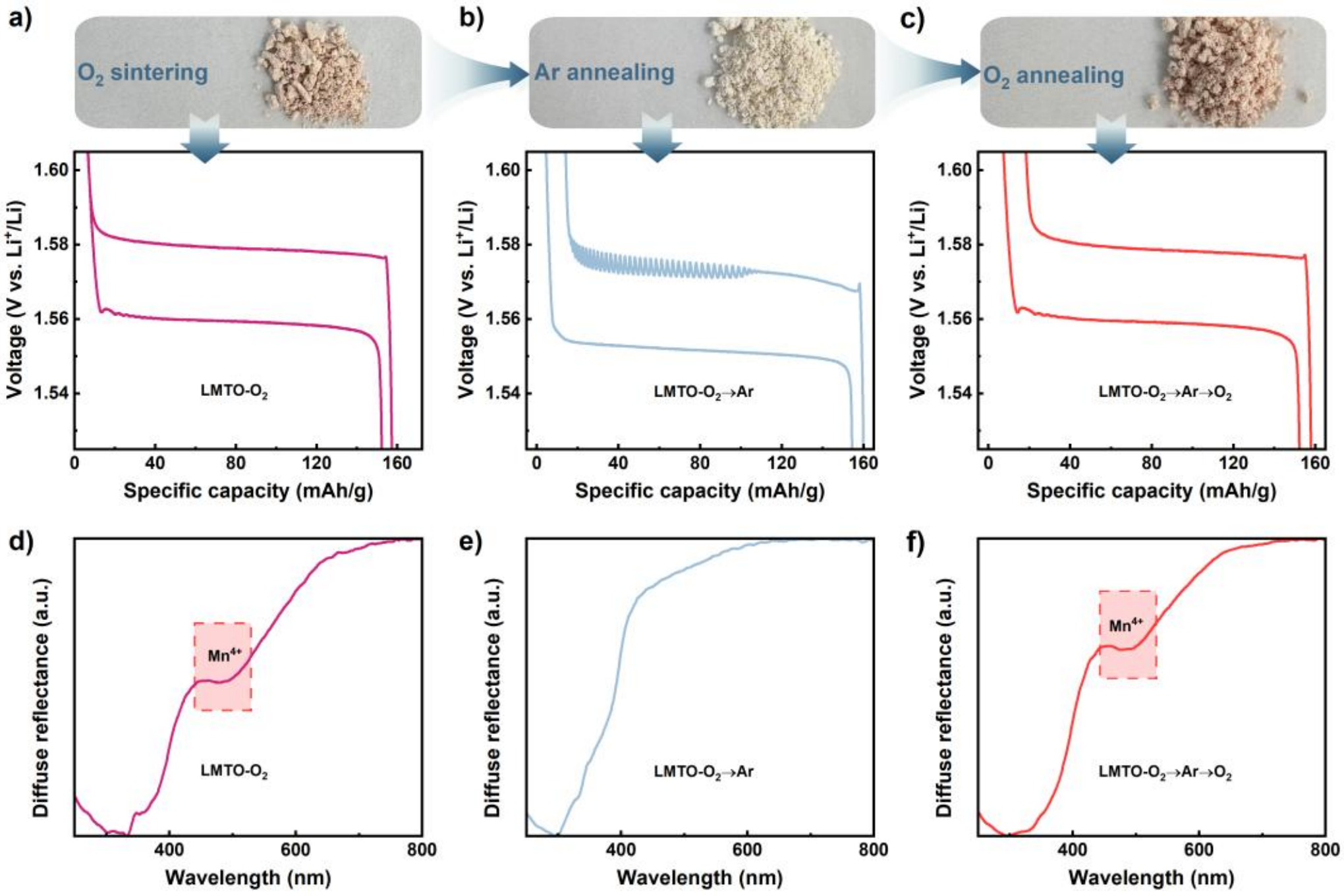

Fig. S10 (a-c) Galvanostatic charge/discharge curves and digital photographs and (d-f) UV-vis DRS of LMTO-O_2, LMTO-O_2→Ar, and LMTO-O_2→Ar→O_2 powder samples. Here, LMTO-O_2→Ar obtained by annealing LMTO-O_2 at 800℃ under Ar atmosphere for 10 h, and LMTO-O_2→Ar→O_2 synthesized by annealing LMTO-O_2→Ar at 800℃ under O_2 atmosphere for 10 h.

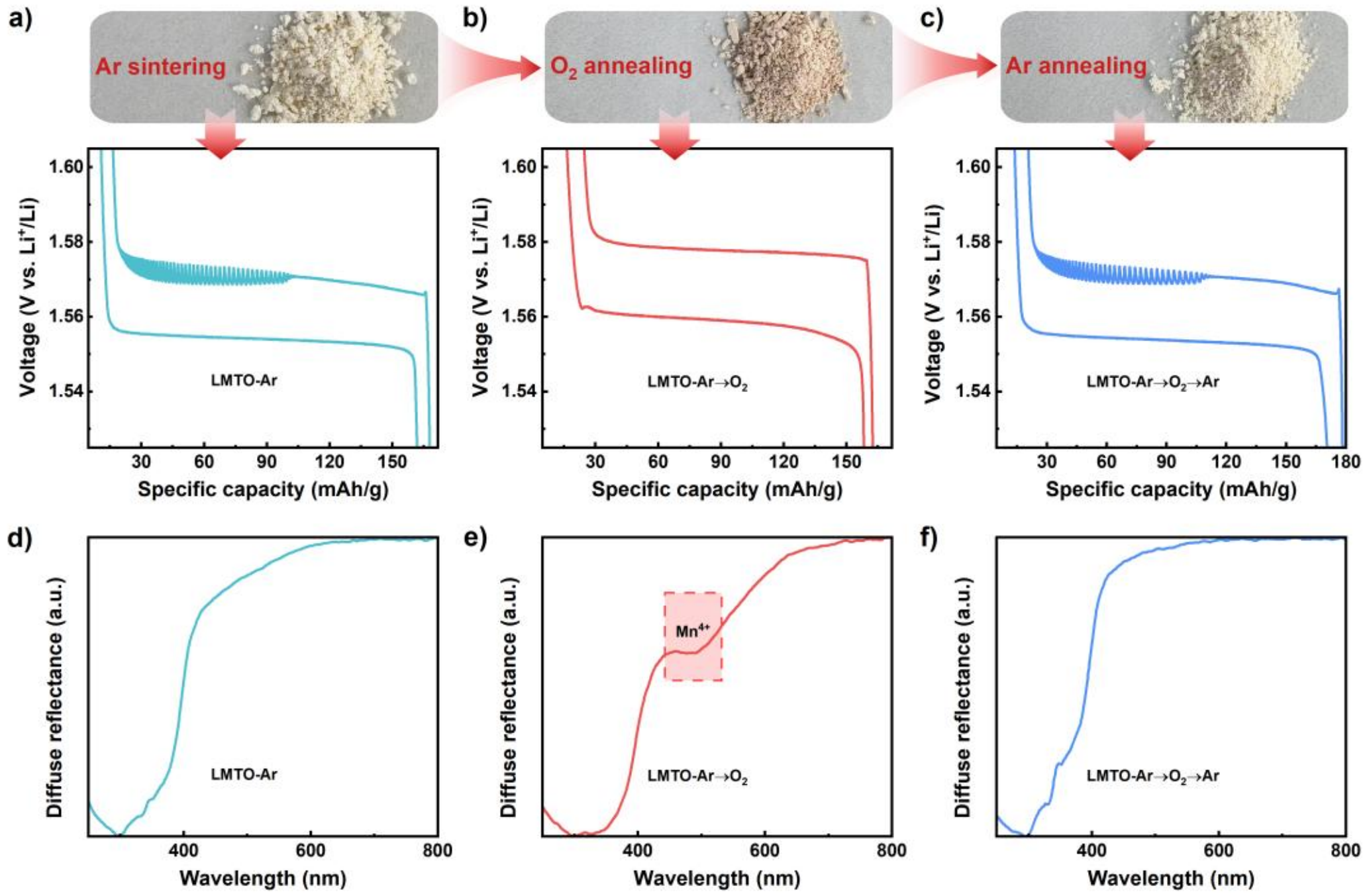

Fig. S11. (a-c) Galvanostatic charge/discharge curves and digital photographs and (d-f) UV-vis DRS of LMTO-Ar, LMTO-Ar→O_2, and LMTO-Ar→O_2→Ar powder samples. Here, LMTO-Ar→O_2, obtained by annealing LMTO-Ar at 800℃ under O_2 atmosphere for 10 h, and LMTO-Ar→O_2→Ar synthesized by annealing LMTO-Ar→O_2 at 800℃ under Ar atmosphere for 10 h.

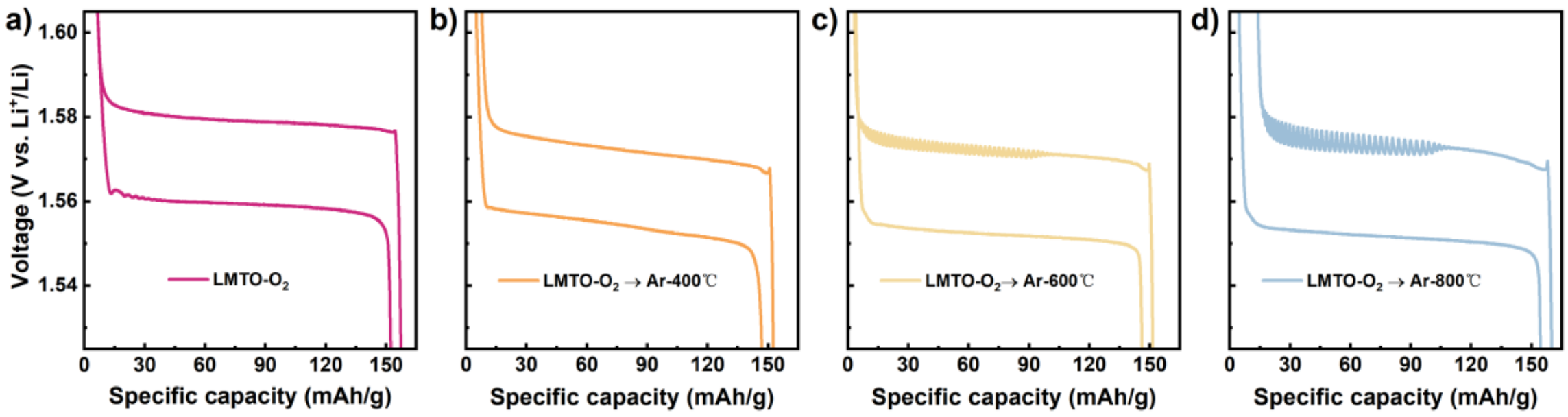

Fig. S12. Galvanostatic charge/discharge curves of (a) LMTO-O_2 and LMTO-O_2 samples by annealing at (b) 400, (c) 600, (d) 800℃ under Ar atmosphere for 10 h, respectively.

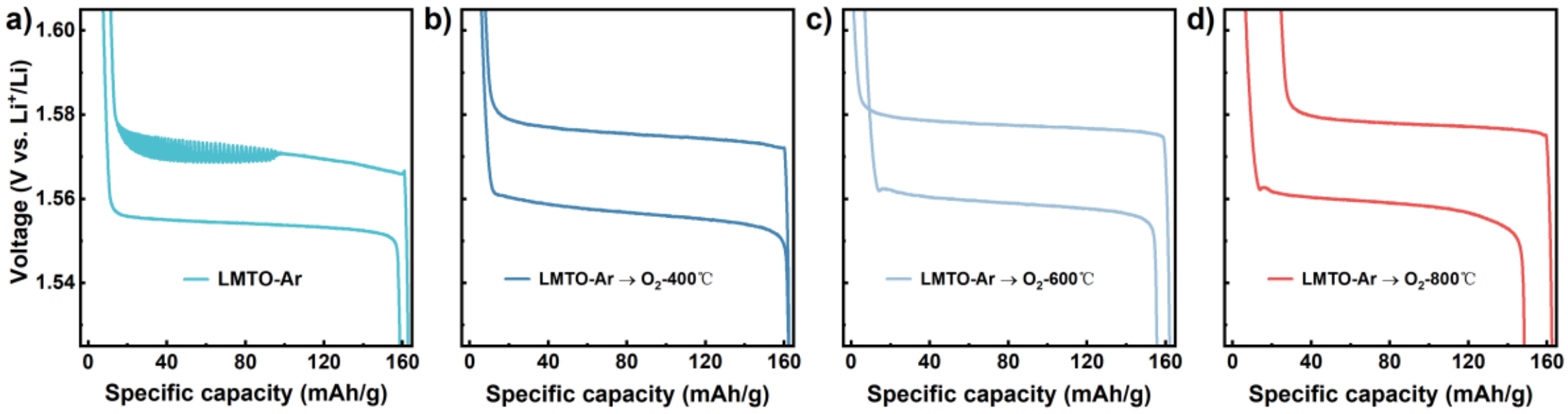

Fig. S13. Galvanostatic charge/discharge curves of (a) LMTO-Ar and LMTO-Ar samples by annealing at (b) 400, (c) 600, (d) 800℃ under O_2 atmosphere for 10 h, respectively.

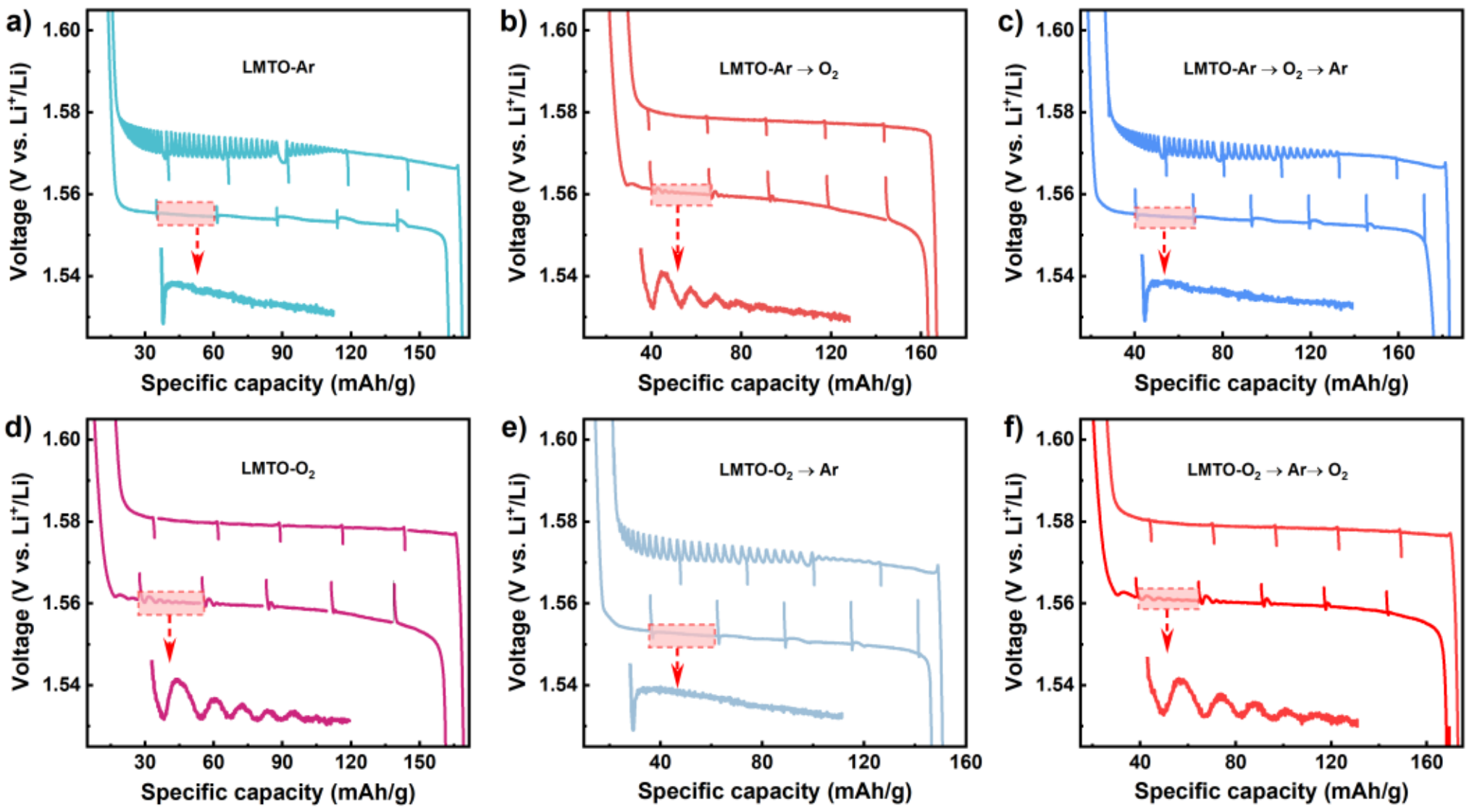

Fig. S14. The discharge-charge profiles by Galvanostatic Intermittent Titration Technique (GITT) for (a) LMTO-Ar, (b) LMTO-Ar→O_2, (c) LMTO-Ar→O_2→Ar, (d) LMTO-O_2 (e) LMTO-O_2→Ar and (f) LMTO-O_2→Ar→O_2 under a current of 0.1C at 25℃. The test procedure contains galvanostatic for 1.5 h and rest for 2 h, which are repeated until reaching the cut-off potential. Inset is the magnification region of corresponding discharge curve.

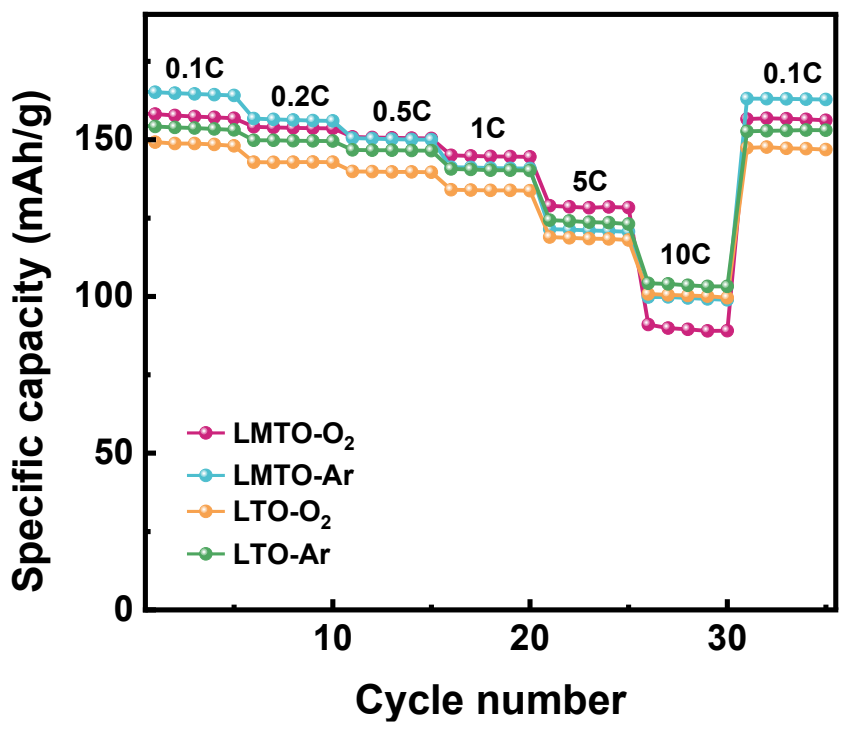

Fig. S15. Rate performance of LTO-O_2, LMTO-O_2, LTO-Ar and LMTO-Ar.

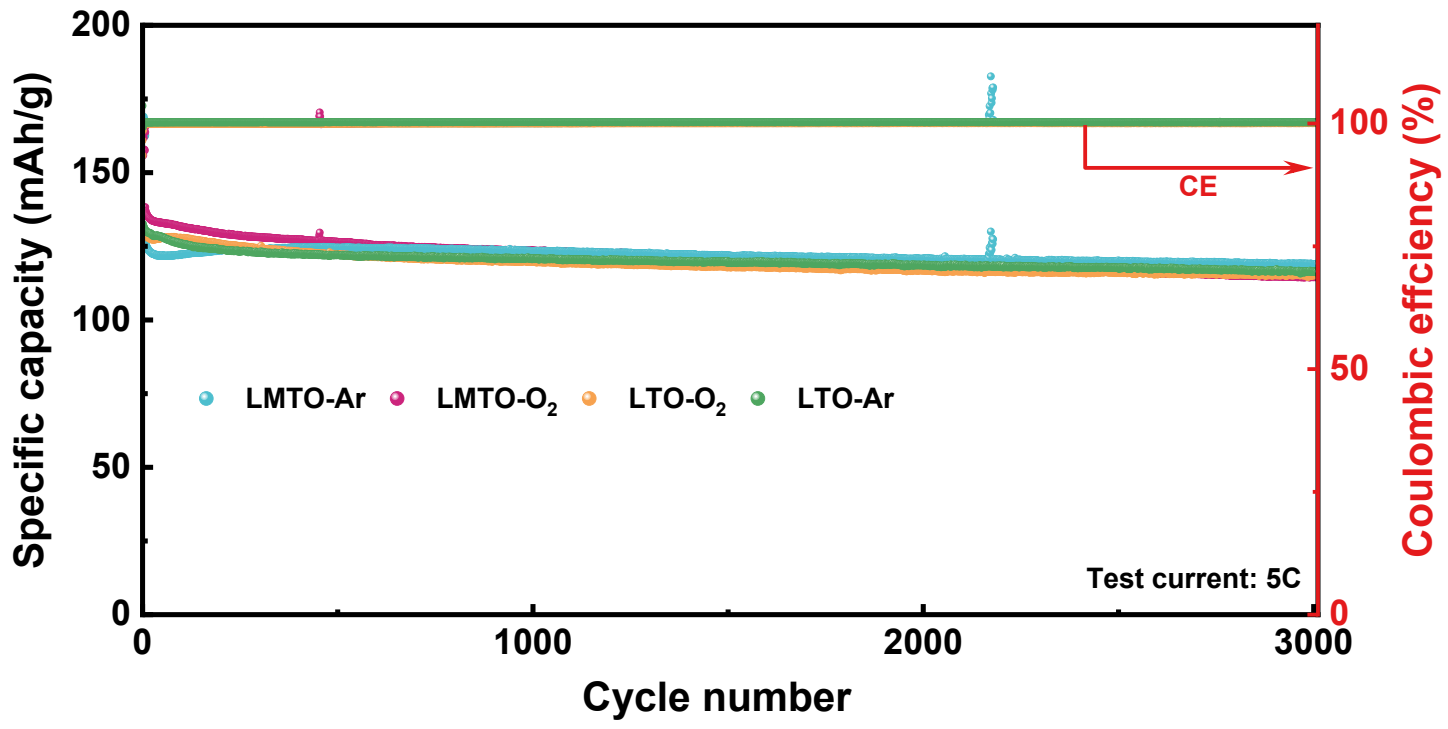

Fig. S16. Cycling performance of LTO-O_2, LMTO-O_2, LTO-Ar and LMTO-Ar at current density of 5C.

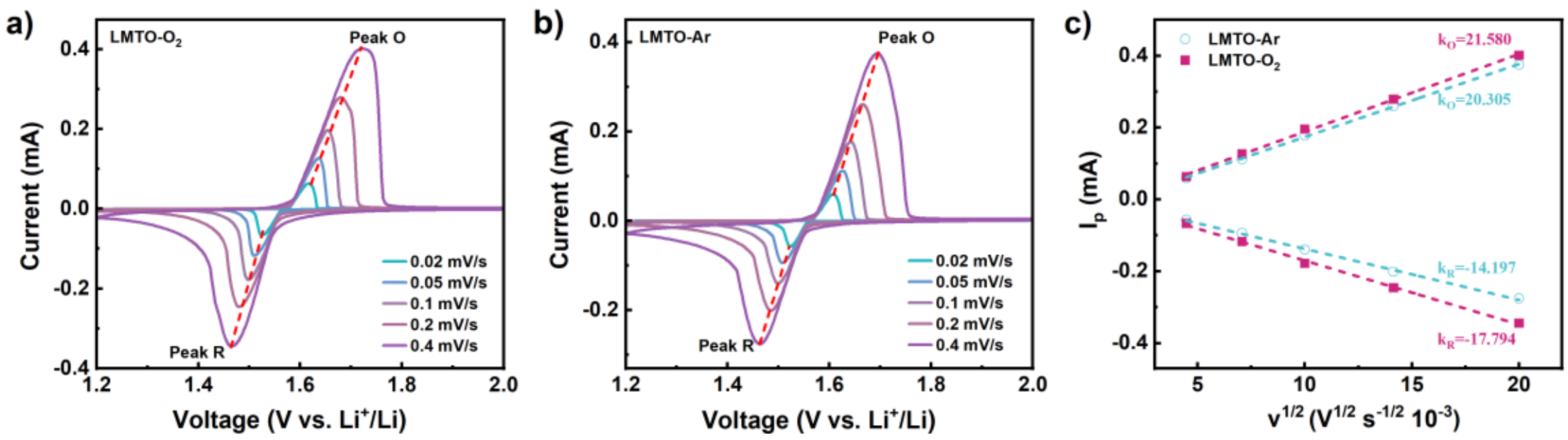

Fig. S17. CV curves of (a) LMTO-O_2 and (b) LMTO-Ar at scan rates from 0.02 to 0.4 mV·s^{-1}. (c) Linear relationship of peak current vs. square root of scan rate, derived from the CV curves for LMTO-O_2 and LMTO-Ar.

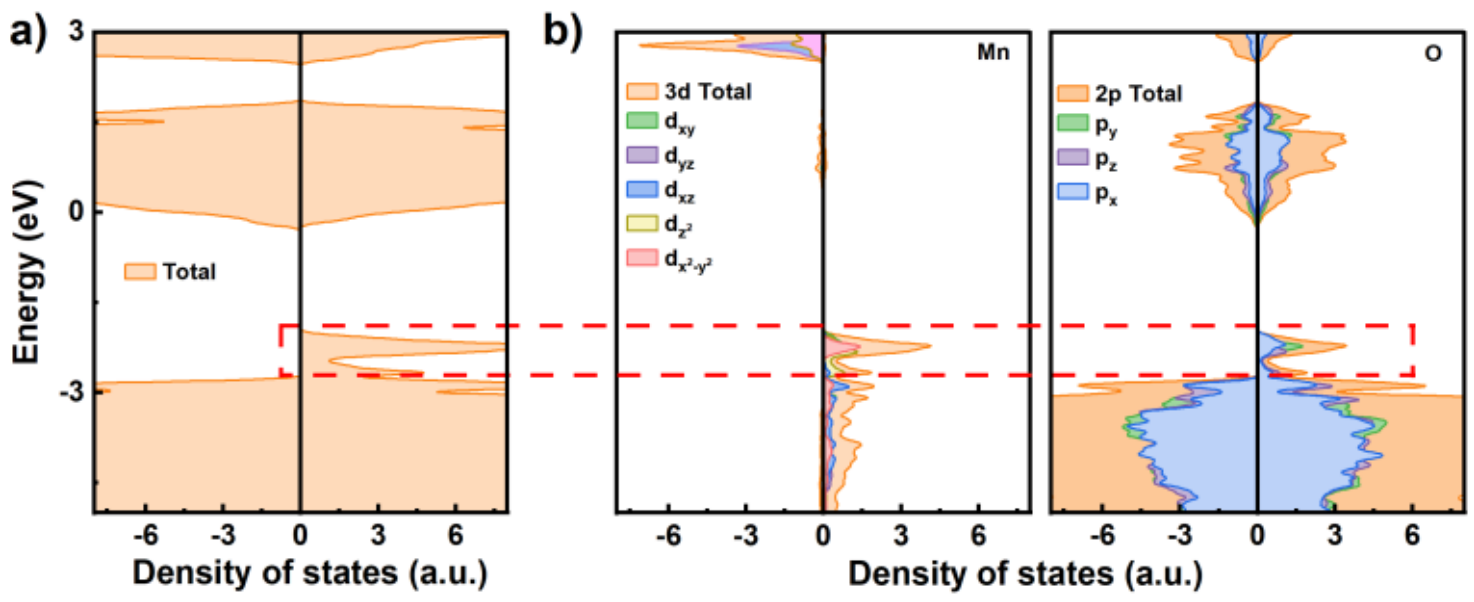

Fig. S18. (a) Total densities of states (TDOS) and (b) projected density of states (PDOS) of Mn 3d and O 2p orbitals for Mn occupied on the 8a Li site.

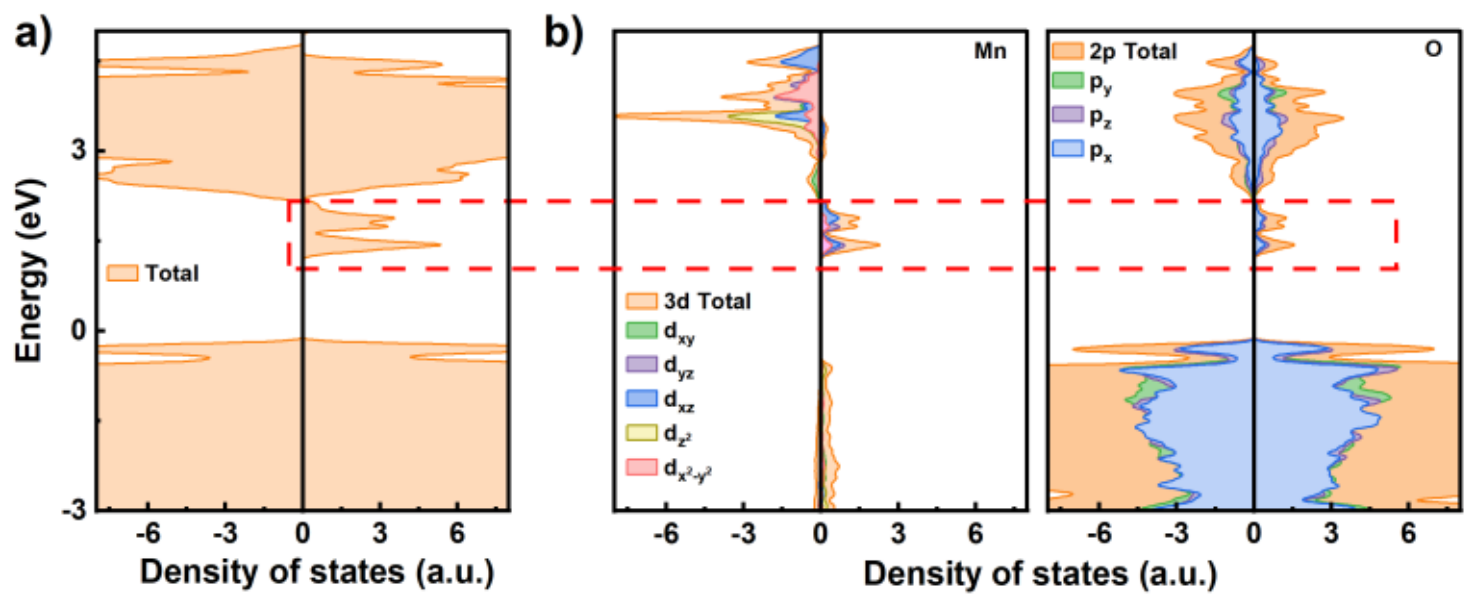

Fig. S19. Total densities of states (TDOS) and (b) projected density of states (PDOS) of Mn 3d and O 2p orbitals for Mn occupied on the 16d Ti site.

Table S1 Experimental parameters of powder X-ray diffraction and refined crystallographic of LMTO-Ar.

Chemical Formula	$Li_{3.8}Mn_{0.05}Ti_5O_{12}$
Radiation type	Cu Kα, λ=1.54060 Å
2θ interval (°)	10.0-120.0
Space group	Fd$\overline{3}$m
a=b=c (Å)	8.360012
V (Å^3)	584.302
R_{wp} [a)]	7.764%
G. O. F	1.24

a) $R_{wp} = \sqrt{\frac{\Sigma w_i |y_i - Y_{C,i}|^2}{\Sigma w_i {y_i}^2}}$

Table S2 Wyckoff positions, atomic coordinates, and occupancies of LMTO-Ar.

Atom	Site	Occupancy	x/a	y/b	z/c
Li	8a	0.9500	-0.1250	-0.1250	-0.1250
Li	16d	0.1670	0.5000	0.5000	0.5000
Mn	8a	0.0175	-0.1250	-0.1250	-0.1250
Ti	16d	0.8330	0.5000	0.5000	0.5000
O	32e	1.0000	0.2640	0.2640	0.2640

Table S3 Experimental parameters of powder X-ray diffraction and refined crystallographic of LMTO-O_2.

Chemical Formula	$Li_{3.8}Mn_{0.05}Ti_5O_{12}$
Radiation type	Cu Kα, λ=1.54060 Å
2θ interval (°)	10.0-120.0
Space group	$Fd\overline{3}m$
a=b=c (Å)	8.35861
V (Å^3)	583.985
R_{wp}	9.496%
G. O. F	1.34

Table S4 Wyckoff positions, atomic coordinates, and occupancies of LMTO-O_2.

Atom	Site	Occupancy	x/a	y/b	z/c
Li	8a	0.9500	-0.1250	-0.1250	-0.1250
Li	16d	0.1670	0.5000	0.5000	0.5000
Mn	16d	0.0080	0.5000	0.5000	0.5000
Ti	16d	0.8250	0.5000	0.5000	0.5000
O	32e	1.0000	0.2640	0.2640	0.2640
